# Total synthesis of TMG-chitotriomycin based on an automated electrochemical assembly of a disaccharide building block

**DOI:** 10.3762/bjoc.13.93

**Published:** 2017-05-16

**Authors:** Yuta Isoda, Norihiko Sasaki, Kei Kitamura, Shuji Takahashi, Sujit Manmode, Naoko Takeda-Okuda, Jun-ichi Tamura, Toshiki Nokami, Toshiyuki Itoh

**Affiliations:** 1 Department of Chemistry and Biotechnology, Graduate School of Engineering, Tottori University, 4-101 Koyama-minami, Tottori 680-8552, Japan; 2 Department of Regional Environment, Faculty of Regional Sciences, Tottori University, 4-101 Koyama-minami, Tottori 680-8551, Japan; 3 Center for Research on Green Sustainable Chemistry, Faculty of Engineering, Tottori University, 4-101 Koyama-minami, Tottori 680-8552, Japan

**Keywords:** automated synthesis, electrochemical oxidation, glycosylation, glucosamine, total synthesis

## Abstract

The total synthesis of TMG-chitotriomycin using an automated electrochemical synthesizer for the assembly of carbohydrate building blocks is demonstrated. We have successfully prepared a precursor of TMG-chitotriomycin, which is a structurally-pure tetrasaccharide with typical protecting groups, through the methodology of automated electrochemical solution-phase synthesis developed by us. The synthesis of structurally well-defined TMG-chitotriomycin has been accomplished in 10-steps from a disaccharide building block.

## Introduction

**Figure 1:**
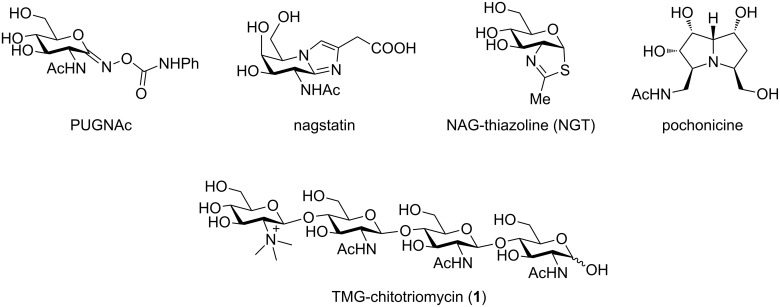
Inhibitors of glucosaminidases.

Degradation of chitin into oligoglucosamines and glucosamine is an important biological process and at least several enzymes such as chitinases and glucosaminidases are involved. Various types of inhibitors such as PUGNAc [1], nagastatin [2], NAG-thiazoline (NGT) [3], and pochonicine [4], have already been developed. These compounds have exhibited strong inhibition activity; however, they have a broad spectrum toward enzymes of various species including animals (Figure 1). *N*,*N*,*N*-Trimethyl-D-glucosaminyl (TMG)-chitotriomycin (**1**) was isolated from *Streptomyces anulatus* by Kanzaki [5–7] and the first total synthesis was completed by Yu [8–9]. Although the activity of TMG-chitotriomycin (**1**) was moderate, it selectively inhibits glucosaminidases derived from insects and fungi. Therefore, TMG-chitotriomycin (**1**) has a potential as a lead compound for safe insecticides and pesticides. Recently, the total synthesis of TMG-chitotriomycin (**1**) initiated from building blocks that were prepared by the degradation of chitin has been reported by Beau [10]; however, practical synthetic methods to provide TMG-chitotriomycin (**1**) and its derivative in preparative scale are still highly desirable.

The automated synthesis of oligosaccharides is a powerful tool for the rapid synthesis of complex oligosaccharides [11–23]. We are interested in the automated synthesis of oligosaccharides based on the concept of “reaction integration” [24] and developed an automated synthesizer for the automated electrochemical assembly of carbohydrate building blocks [25]. During the course of our study, we have achieved the synthesis of a potential precursor of TMG-chitotriomycin (**1**); however, we obtained the tetrasaccharide as a mixture of α- and β-isomers in the terminal glycosidic linkage [26]. Here we report the total synthesis of TMG-chitotriomycin (**1**) as a single stereoisomer, which was prepared by automated electrochemical assembly started from a disaccharide building block.

## Results and Discussion

**Scheme 1:**
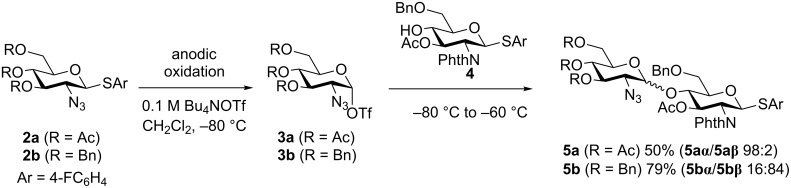
Synthesis of disaccharide donors.

To synthesize the potential precursor **7** of TMG-chitotriomycin (**1**) stereoselectively, we initiated our study by optimization of the reaction conditions of the first glycosylation using 2-deoxy-2-azidothioglycoside **2** as a glycosyl donor. The azido group at the C2-position is a well-known substituent, which facilitates the formation of an α-glycosidic linkage selectively due to the lack of neighboring group participation [27]. 4-Fluorophenyl 3,4,6-tri-*O*-acetyl-2-deoxy-2-azido-β-D-thioglucoside (**2a**) afforded the corresponding disaccharide α-isomer **5aα** exclusively by the reaction with building block **4** via the glycosyl triflate intermediate **3a** (Scheme 1). On the other hand, 4-fluorophenyl 3,4,6-tri-*O*- benzyl-2-deoxy-2-azido-β-D-thioglucoside (**2b**) gave the disaccharide β-isomer **5bβ** as the major product. Although the disaccharide α- and β-isomers of **5b** (**5bα/5bβ**) have the same retention factor (*R*_f_) in thin-layer chromatography (TLC, *R*_f_ = 0.40, hexane/EtOAc 5:2 as an eluent), the pure β-disaccharide isomer **5bβ** was obtained as colorless crystals after careful separation by silica gel chromatography.

**Figure 2:**
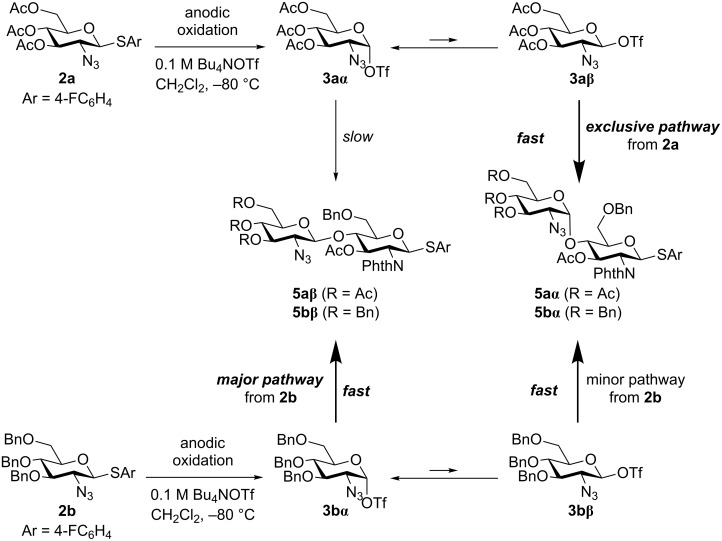
Proposed mechanism and origin of the selectivity.

It is still not clear why the disaccharide β-isomer **5bβ** was obtained as a major product from glycosyl triflate **3**. We now assume that the difference in the reactivity of the α- and the β-glycosyl triflate intermediates **3** might determine the observed selectivity (Figure 2). We previously established that glycosyl triflate **3a** was derived from thioglycoside **2a** by an NMR study under low-temperature conditions, in which the glycosyl triflate α-isomer **3aα** was confirmed as an exclusive chemical species [28–29]. Taking the stability of the glycosyl triflate α-isomer **3aα** into consideration, we propose a reaction mechanism involving α/β isomerization of glycosyl triflate **3a** as shown in Figure 2. The more reactive β-isomer **3aβ** might give the disaccharide α-isomer **5aα** exclusively if there is an equilibrium between the α-isomer and the β-isomer of **3a**. To the contrary, glycosyl triflate **3b**, derived from thioglycoside **2b**, might be more reactive and affords the β-product **5bβ** before isomerization from the α-isomer **3bα** to the β-isomer **3bβ**. In this case, glycosylation via **3bα** becomes the major pathway [30]. Although it is hard to exclude another reaction mechanism involving oxocarbenium ions as reactive intermediates, the commonly accepted reactivity difference between α- and β-isomers of glycosyl triflate **3** seems to explain the observed selectivity well.

**Figure 3:**
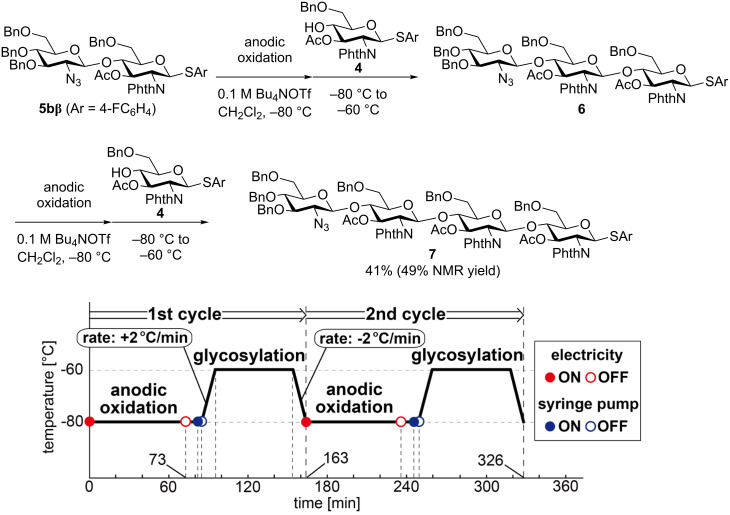
Synthesis of TMG-chitotriomycin precursor **7**.

Next, we attempted to synthesize the potential precursor **7** of TMG-chitotriomycin (**1**) using disaccharide **5bβ** as a building block as illustrated in Figure 3. The automated electrochemical assembly of building blocks was initiated by the anodic oxidation of **5bβ** and the subsequent coupling with thioglycoside **4** afforded the corresponding trisaccharide **6** as an intermediate after the 1st cycle. The same process was repeated automatically in the 2nd cycle and target tetrasaccharide **7** was obtained in 41% yield after purification by preparative gel permeation chromatography (GPC).

**Figure 4:**
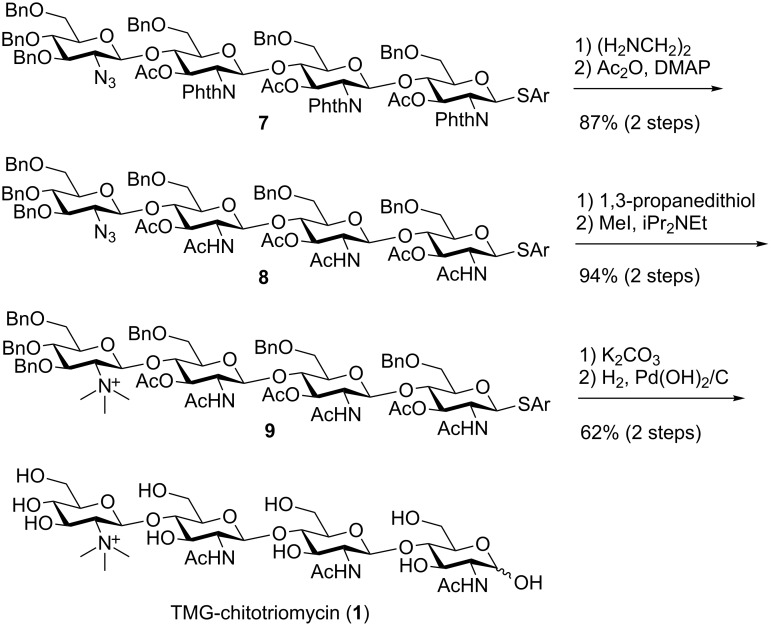
Synthess of TMG-chitotriomycin (**1**).

Deprotection and introduction of the TMG part to tetrasaccharide **7** were achieved by following the procedure reported by Yu and co-workers (Figure 4) [8]. Phthaloyl groups and acetyl groups of **7** are removed by the reaction with ethylenediamine followed by applying the conventional acetylation protocol with acetic anhydride (Ac_2_O) in the presence of *N*,*N*-dimethylaminopyridine (DMAP) to convert the amino groups into acetamide groups and to protect the hydroxy groups as acetyl groups. The 2-azido group of tetrasaccharide **8** was then reduced to a 2-amino group with 1,3-propanedithiol. Thus-obtained tetrasaccharide was treated with iodomethane (MeI) and *N*,*N*-diisopropylethylamine (iPr_2_NEt) to prepare the TMG part of tetrasaccharide **9**. Deprotection of acetyl groups at the 3-*O*-positions and the subsequent global deprotection of the benzyl groups and the anomeric thioaryl group of the tetrasaccharide by hydrogenation with hydrogen gas in the presence of a palladium catalyst afforded TMG-chitotriomycin (**1**) in 21% yield (10 steps from disaccharide **5bβ**) [31].

## Conclusion

In conclusion, we have achieved the stereoselective synthesis of TMG-chitotriomycin (**1**) based on the automated electrochemical assembly of disaccharide and monosaccharide building blocks. Thus-obtained structurally well-defined tetrasaccharide gave TMG-chitotriomycin after manipulations of the amino groups and global deprotection. Further investigations to improve the β-selectivity in the disaccharide synthesis and a large scale synthesis are in progress in our laboratory.

## Experimental

### General

^1^H and ^13^C NMR spectra were recorded in CDCl_3_ on a Bruker AVANCE II 600 spectrometer (^1^H 600 MHz, ^13^C 150 MHz) with Me_4_Si as an internal standard unless otherwise noted. Mass spectra were obtained on a Thermo Scientific Exactive mass spectrometer. Thin-layer chromatography (TLC) was carried out by using Merck precoated silica gel F254 plates (thickness 0.25 mm). Flash chromatography was carried out on a column of silica gel (Kanto Chem. Co., Silica Gel N, spherical, neutral). Gel permeation chromatography (GPC) was carried out on a Japan Analytical Industry LC-918 equipped with JAIGEL-2H using CHCl_3_ as eluent. All reactions were performed under an Ar atmosphere unless otherwise noted.

### Materials

All materials including solvents were purchased and used without further purification. Carbohydrate building blocks **2a**, **2b**, and **4** were prepared according to an previous report [11].

### Automated synthesis of TMG-chitotriomycin precursor **7**

The H-type glass cell equipped with glass filter was dried under vacuum and then filled with argon gas. Disaccharide building block **5bβ** (0.43 mmol, 436 mg) was placed in the anodic chamber together with Bu_4_NOTf (1.7 mmol, 672 mg) and anhydrous dichloromethane (16 mL). In the cathodic chamber Bu_4_NOTf (1.7 mmol, 676 mg) and anhydrous dichloromethane (16 mL) was placed with TfOH (1 mmol, 90 μL). The automated synthesis was started immediately after the cooling bath temperature reached −80 °C. The anodic oxidation (1.05 F/mol, 10 mA) takes 73 minutes and then a dichloromethane solution containing building block **4** (0.43 mmol, 1.0 mL) was added by a syringe pump. After addition of the building block, the temperature of the cooling bath was raised to −60 °C and the reaction mixture was stirred for 1 h at this temperature. Then the temperature of the cooling bath was cooled again at −80 °C to start the 2nd cycle. It takes about 326 minutes (ca. 5 h 30 min) to complete the automated assembly of building blocks. The reaction was quenched with Et_3_N (0.5 mL) at −80 °C and the cell was taken from the cooling bath. The reaction mixture of the anodic chamber was evaporated and the thus-obtained crude product was purified by silica gel chromatography (hexane/EtOAc 1:1 as eluent). The precursor **7** was obtained in 49% yield (411 mg) together with building block **4** as an impurity. Further purification by GPC (CHCl_3_ as eluent) afforded **7** in 41% yield (328 mg, 0.178 mmol).

## Supporting Information

File 1 Experimental details of electrochemical glycosylation, global deprotection, and NMR spectra of unknown compounds.
