# Supplementary material for: Total synthesis of TMG-chitotriomycin based on an automated electrochemical assembly of a disaccharide building block
Source: Beilstein J Org Chem. 2017 May 16;13:919–24. doi: 10.3762/bjoc.13.93 (PMC5480352; doi:10.3762/bjoc.13.93)
Supplement: File 1 — Experimental details of electrochemical glycosylation, global deprotection, and NMR spectra of unknown compounds. [file Beilstein_J_Org_Chem-13-919-s001.pdf]

Supporting Information

for

Total synthesis of TMG-chitotriomycin based on automated electrochemical assembly of a disaccharide building block

Yuta Isoda[1], Norihiko Sasaki[1], Kei Kitamura[1], Shuji Takahashi[1], Sujit Manmode[1], Naoko Takeda-Okuda[2], Jun-ichi Tamura[1,2,3], Toshiki Nokami[1,3]* and Toshiyuki Itoh[1,3]*

Address: [1]Department of Chemistry and Biotechnology, Graduate School of Engineering, Tottori University, 4-101 Koyama-minami, Tottori 680-8552, Japan, [2]Department of Regional Environment, Faculty of Regional Sciences, Tottori University, 4-101 Koyama-minami, Tottori 680-8551, Japan and [3]Center for Research on Green Sustainable Chemistry, Faculty of Engineering, Tottori University, 4-101 Koyama-minami, Tottori 680-8552, Japan

Experimental details of electrochemical glycosylation, global deprotection, and NMR spectra of unknown compounds

Contents

1.	General	S2
2.	Synthesis of disaccharide donors	S2–S3
3.	Deprotection of the precursor of TMG-chitotriomycin	S3–S5
4.	References	S6
5.	^{1}H, ^{13}C NMR, H,H-COSY, and HMQC spectra of disaccharides **5aα** and **5bα**	S7–S10
6.	^{1}H, ^{13}C-NMR, H,H-COSY, and HMQC spectra of tetrasaccharides **8** and **9**	S11–S14

1. General

^{1}H and ^{13}C NMR spectra were recorded on a Bruker AVANCE II 600 (^{1}H 600 MHz, ^{13}C 150 MHz). Electro-spray ionization mass spectra (ESI–TOF MS) were recorded on Thermo Scientific Exactive spectrometer. Sephadex LH-20 was used for gel filtration chromatography and Kanto silica gel (spherical, neutral, 63–210 μm) was used for column chromatography. Optical rotation was recorded on JASCO DIP-370 digital polarimeter in chloroform. Merck TLC (silica gel 60 F_{254}) was used for TLC analysis. Carbohydrate building blocks **2a**,[1] **2b**,[1] and **4**[1] were prepared according to the reported procedures. NMR spectra of **5bβ** have already provided in our previous report. [1] TMG-chitotriomycin (**1**) thus-obtained was compared with that synthesized in previous reports.[2,3] Unless otherwise noted, all materials were obtained from commercial suppliers and used without further purification.

2. Synthesis of disaccharide donors

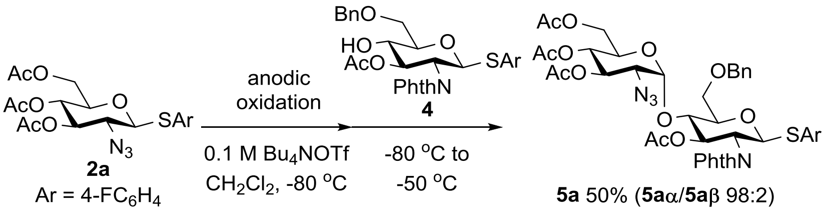

Scheme S1.

The synthesis of disaccharide glycosyl donor **5a** was carried out in an H-type divided cell (4G glass filter) equipped with a carbon felt anode (Nippon Carbon JF-20-P7) and a platinum plate cathode (20 mm × 20 mm). In the anodic chamber was placed thioglycoside **2a** (134 mg, 0.25 mmol) in anhydrous dichoromethane (10 mL). In the cathodic chamber was placed TfOH (22 μL, 0.2 mmol) and in anhydrous dichoromethane (10 mL). The constant current electrolysis (8.0 mA) was carried out at −80 °C with magnetic stirring until 1.0 F/mol of electricity was consumed. After the electrolysis, **4** (165 mg, 0.30 mmol) in anhydrous dicloromethane (1.0 mL) was added and stirred for 1 h at −60 °C. Then Et_3N (0.3 mL) was added to the both chambers and the mixture was stirred at ambient temperature. After removal of solvent the crude product was purified with silica gel chromatography (hexane/EtOAc = 1:1) to afford disaccharide **5aα** as a major product (204 mg, 59% NMR yield) together with **4**. Further purification with preparative GPC gave disaccharide **5a** in 50% yield (109 mg, 0.126 mmol, **5aα**/**5aβ** = 98:2). **4-Fluorophenyl 3,4,6-tri-*O*-acetyl-2-deoxy-2-azido-α-D-glucopyranosyl-(1→4)-3-*O*-acetyl-6-*O*-benzyl-2-deoxy-2-phthalimido-1-thio-β-D-glucopyranoside (5aα)**. TLC (Hexane/EtOAc = 1:1): R_f 0.55. $[\alpha]_D$ = +66.0 (*c* = 1.0, $CHCl_3$, 25 °C). ^{1}H NMR ($CDCl_3$, 600 MHz) δ 7.88 (d, *J* = 7.2 Hz, 1 H), 7.84 (d, *J* = 6.0 Hz, 1 H), 7.74 (dd, *J* = 5.4, 3.6 Hz, 2 H), 7.41 (dd, *J* = 9.0, 5.4 Hz, 2 H), 7.39 – 7.30 (m, 5 H), 6.90 (pseudo-t, *J* = 9.0 Hz, 2 H), 5.79 (dd, *J* = 9.6, 9.0 Hz, 1 H), 5.68 (d, *J* = 10.8 Hz, 1 H), 5.32 (dd, *J* = 10.2, 9.0 Hz, 1 H), 5.22 (d, *J* = 3.6 Hz, 1 H), 4.97 (pseudo-t, *J* = 10.2 Hz, 1 H), 4.62 (s, 2 H), 4.17 (pseudo-t, *J* = 10.2 Hz, 1 H), 4.16 (dd, *J* = 9.0, 3.6 Hz, 1 H), 4.01 (pseudo-t, *J* = 9.6 Hz, 1 H), 3.99 (dt, *J* = 10.8, 1.8 Hz, 1 H), 3.86 (dd, *J* = 11.4, 4.2 Hz, 1 H), 3.82 (d, *J* = 10.8 Hz, 1 H), 3.81 (d, *J* =12.6 Hz, 1 H), 3.79 (ddd, *J* = 10.2, 4.2, 1.8 Hz, 1 H), 3.38 (dd, *J* = 10.8, 3.6 Hz, 1 H), 2.03 (s, 3 H),

2.02 (s, 3 H), 2.01 (s, 3 H), 1.86 (s, 3 H). ^{13}C NMR ($CDCl_3$, 150 MHz) δ 170.3, 169.7, 169.6, 169.5, 167.8, 167.2, 163.1 (d, *J* = 247.8 Hz), 137.8, 136.3 (d, *J* = 8.6 Hz), 134.5, 134.2, 131.6, 131.0, 128.4, 127.8, 125.3 (d, *J* = 3.2 Hz), 123.7, 123.5, 115.9 (d, *J* = 21.8 Hz), 98.2, 82.4, 78.3, 75.1, 74.1, 73.5, 70.2, 68.7, 68.3, 68.0, 61.3, 60.9, 53.9, 20.6, 20.53, 20.50, 20.4. HRMS (ESI) *m/z* calcd for $C_{41}H_{41}FN_4NaO_{14}S$ $[M+Na]^+$, 887.2216; found, 887.2214.

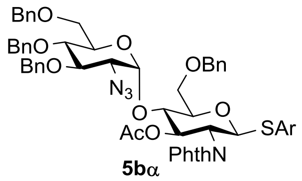

5bα

Ar = 4-FC_6H_4

Building blocks **2b** (146 mg, 0.25 mmol) and **4** (165 mg, 0.30 mmol) afforded disaccharide **5b** as a white solid in 79% yield (α/β = 16:84, 0.198 mmol).[1] Silica gel chromatography (hexane/EtOAc = 4:1 as eluent) was repeated for 4 times to obtain α-isomer **5bα** as a pure product (10 mg). **4-Fluorophenyl 3,4,6-tri-*O*-benzyl-2-deoxy-2-azido-α-D-glucopyranosyl-(1→4)-3-*O*-acetyl-6-*O*-benzyl-2-deoxy-2-phthalimido-1-thio-β-D-glucopyranoside (5bα)**. TLC (Hexane/EtOAc 5:2): R_f 0.34. $[\alpha]_D$ = +21.8 (*c* = 1.2, $CHCl_3$, 25 °C). ^{1}H NMR ($CDCl_3$, 600 MHz) δ 7.89 – 7.85 (m, 2 H), 7.77 – 7.73 (m, 2 H), 7.42 (dd, *J* = 8.4, 5.4 Hz, 2 H), 7.39 – 7.25 (m, 18 H), 7.14 – 7.13 (m, 2 H), 6.88 (pseudo-t, *J* = 8.4 Hz, 2 H), 5.79 (pseudo-t, *J* = 9.0 Hz, 1 H), 5.68 (d, *J* = 10.2 Hz, 1 H), 5.15 (d, *J* = 3.6 Hz, 1 H), 4.83 (d, *J* = 10.8 Hz, 1 H), 4.79 (d, *J* = 10.8 Hz, 1 H), 4.76 (d, *J* = 11.4 Hz, 1 H), 4.55 (s, 2 H), 4.53 (d, *J* = 12.6 Hz, 1 H), 4.47 (d, *J* = 10.8 Hz, 1 H), 4.36 (d, *J* = 12.0 Hz, 1 H), 4.18 (pseudo-t, *J* = 10.2 Hz, 1 H), 3.95 (pseudo-t, *J* = 9.0 Hz, 1 H), 3.84 (d, *J* = 9.6 Hz, 1 H), 3.83 – 3.79 (m, 2 H), 3.78 – 3.74 (m, 2 H), 3.67 (pseudo-t, *J* = 9.6 Hz, 1 H), 3.61 (dd, *J* = 10.8, 3.0 Hz, 1 H), 3.44 (d, *J* = 10.2 Hz, 1 H), 3.36 (dd, *J* = 10.2, 3.6 Hz, 1 H), 1.87 (s, 3 H). ^{13}C NMR ($CDCl_3$, 150 MHz) δ 170.1, 168.0, 167.3, 163.1 (d, *J* = 247.2 Hz), 138.2, 137.8, 137.71, 137.68, 136.2 (d, *J* = 8.4 Hz), 134.5, 134.2, 131.8, 131.2, 128.47, 128.43, 128.41, 128.40, 128.1, 127.9, 127.85, 127.80, 127.76, 127.65, 127.4, 125.7 (d, *J* = 3.5 Hz), 123.7, 123.5, 116.0 (d, *J* = 21.8 Hz), 99.2, 82.4, 80.0, 78.6, 78.0, 75.6, 75.5, 75.0, 74.4, 73.5, 73.4, 71.8, 69.1, 67.9, 63.6, 54.0, 20.5. HRMS (ESI) *m/z* calcd for $C_{56}H_{53}FN_4NaO_{11}S$ $[M+Na]^+$, 1031.3308; found, 1031.3256.

3. Deprotection of the precursor of TMG-chitotriomycin

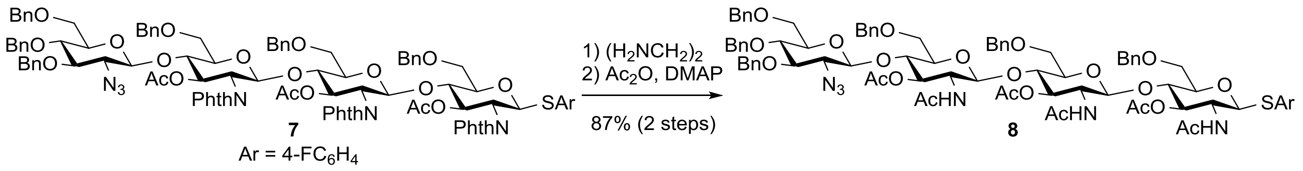

Scheme S3.

Tetrasaccharide **7** (194 mg, 0.105 mmol) was dissolved in ethanol (5 mL). Then ethylenediamine (15.6 mmol, 1.0 mL) was added to the reaction mixture and stirred at room temperature for 10 min and reflux for

12 h. The solvent was removed under reduced pressure and dried under vacuum. To the pyridine (5 mL) solution of thus-obtained product and *N*,*N*-dimethylaminopyridine (0.26 mmol, 30.5 mg) acetic anhydride (18.1 mmol, 1.71 mL) was added and stirred at room temperature for 12 h. The reaction was quenched by addition of CH_2Cl_2 (10 mL) and the organic layer was washed with 1 M aqueous HCl solution and saturated aqueous $NaHCO_3$ solution, respectively. The organic layer was dried over Na_2SO_4 and the solvent was removed under reduced pressure. Thus-obtained crude product was purified with silica gel chromatography (CH_2Cl_2/MeOH 60:1 to 20:1) to obtain tetrasaccharide **8** (145 mg, 0.91 mmol) as a white solid in 87% yield (2 steps). **4-Fluorophenyl 3,4,6-tri-*O*-benzyl-2-deoxy-2-azido-β-D-glucopyranosyl-(1→4)-3-*O*-acetyl-6-*O*-benzyl-2-deoxy-2-acetamide-β-D-glucopyranosyl-(1→4)-3-*O*-acetyl-6-*O*-benzyl-2-deoxy-2-acetamide-β-D-glucopyranosyl-(1→4)-3-*O*-acetyl-6-*O*-benzyl-2-deoxy-2-acetamide-1-thio-β-D-glucopyranoside (8)**. TLC (EtOAc): R_f 0.25. $[\alpha]_D$ = -51.6 (*c* = 1.0, $CHCl_3$, 25 °C). ^{1}H NMR ($CDCl_3$, 600 MHz) δ 7.53 – 7.48 (m, 4 H), 7.45 – 7.37 (m, 4 H), 7.36 – 7.24 (m, 24 H), 7.15 – 7.12 (m, 2 H), 6.96 (pseudo-t , *J* = 9.0 Hz, 2 H), 6.05 – 5.99 (bs, 1 H), 5.18 (d, *J* = 9.0 Hz, 1 H), 4.96 (pseudo-t, *J* = 9.0 Hz, 1 H), 4.81 (d, *J* = 10.8 Hz, 1 H), 4.76 (d, *J* = 12.0 Hz, 2 H), 4.75 (d, *J* = 11.4 Hz, 2 H), 4.71 – 4.68 (m, 2 H), 4.65 (pseudo-t, *J* = 9.6 Hz, 1 H), 4.52 (pseudo-t, *J* = 12.0 Hz, 3 H), 4.47 – 4.45 (m, 3 H), 4.44 – 4.37 (m, 4 H), 4.23 (d, *J* = 12.0 Hz, 1 H), 4.20 (d, *J* = 8.4 Hz, 1 H), 4.16 (d, *J* = 9.6 Hz, 1 H), 4.13 (d, *J* = 7.8 Hz, 1 H), 4.08 (d, *J* = 8.4 Hz, 1 H), 3.93 (d, *J* = 9.6 Hz, 1 H), 3.91 – 3.74 (m, 5 H), 3.67 – 3.46 (m, 8 H), 3.31 – 3.26 (m, 1 H), 3.27 (dd, *J* =9.6, 8.4 Hz, 1 H), 3.19 (dt, *J* = 9.0, 2.4 Hz, 1 H), 3.16 (pseudo-t, *J* = 9.6 Hz, 1 H), 3.03 (dt, *J* = 4.8, 1.8 Hz, 1 H), 2.05 (s, 3 H), 2.00 (s, 3 H), 1.93 (s, 3 H), 1.92 (s, 3 H), 1.68 (s, 3 H), 1.67 (s, 3 H). ^{13}C NMR ($CDCl_3$, 150 MHz) δ 171.3, 171.2, 170.0, 169.8, 169.5, 162.6 (d, *J* = 246.3 Hz), 138.0, 137.9, 137.6, 137.5, 137.4, 137.0, 134.7 (d, *J* = 8.0 Hz), 129.33, 129.29, 129.1, 128.8, 128.6, 128.5, 128.4, 127.9, 127.8, 127.77, 127.71, 127.69, 127.6, 115.8 (d, *J* = 21.6 Hz), 101.0, 100.6, 86.8, 83.0, 78.6, 75.2, 74.7, 74.4, 74.2, 74.0, 73.9, 73.7, 73.5, 73.1, 72.9, 68.3, 67.9, 66.9, 66.3, 53.7, 52.3, 23.2, 23.1, 23.0, 20.7, 20.51, 20.46. HRMS (ESI) *m/z* calcd for $C_{84}H_{95}FKN_6O_{22}S$ $[M+K]^+$, 1629.5836; found, 1629.5841.

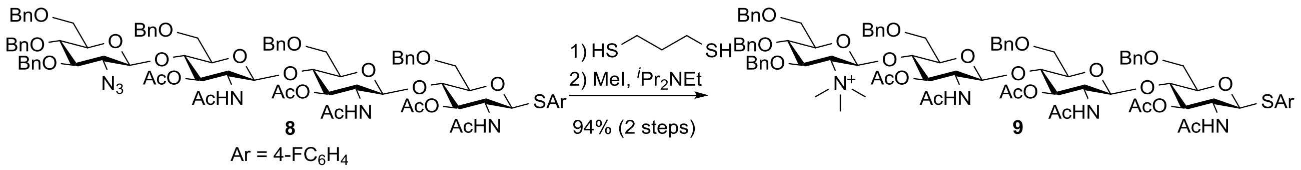

Scheme S4.

Tetrasaccharide **8** (145 mg, 0.091 mmol) was dissolved in a mix-solvent of pyridine and water (5.0 mL/1.3 mL). Then Et_3N (0.4 mL) and 1,3-propanedithiol (7.5 mmol, 0.75 mL) were successively added to the reaction mixture and stirred at room temperature for 12 h. The completion of the reaction was confirmed by ESIMS analysis and the solvent was removed under reduced pressure. The crude product was purified by silica gel chromatography (CH_2Cl_2/MeOH/Et_3N = 80:2:1 as eluent) and dried under vacuum. To the THF (7.4 mL) solution of thus-obtained product *N*,*N*-diisopropylamine (4.3 mmol, 0.75 mL) and methyl iodide (80.5 mmol, 5.0 mL) were added and stirred at room temperature for 12 h. The reaction was quenched by addition of EtOAc (20 mL) and evaporated to remove

solvent. The crude product was purified with silica gel chromatography (CH_2Cl_2/MeOH 8:1) to obtain tetrasaccharide **9** (138 mg, 0.86 mmol) in 94% yield (2 steps) **4-Fluorophenyl 3,4,6-tri-*O*-benzyl-2-deoxy-2-trimethylammonium-β-D-glucopyranosyl-(1→4)-3-*O*-acetyl-6-*O*-benzyl-2-deoxy-2-acetamide-β-D-glucopyranosyl-(1→4)-3-*O*-acetyl-6-*O*-benzyl-2-deoxy-2-acetamide-β-D-glucopyranosyl-(1→4)-3-*O*-acetyl-6-*O*-benzyl-2-deoxy-2-acetamide-1-thio-β-D-glucopyranoside (9)**. TLC (MeOH/EtOAc 1:1): R_f 0.50. $[\alpha]_D$ = -34.4 (*c* = 1.0, $CHCl_3$, 25 °C). ^{1}H NMR ($CDCl_3$, 600 MHz) δ 7.52 (dd, *J* = 5.4, 3.6 Hz, 2 H), 7.43 – 7.34 (m, 8 H), 7.34 – 7.22 (m, 16 H), 7.22 – 7.16 (m, 4 H), 7.04 (d, *J* = 6.6 Hz, 2 H), 6.93 (pseudo-t , *J* = 8.4 Hz, 2 H), 6.46 (bs, 1 H), 5.43 (bs, 1H), 5.20 (bs, 1 H), 5.13 (d, *J* = 6.6 Hz, 1 H), 5.06 (pseudo-t, *J* = 9.0 Hz, 1 H), 4.87 (pseudo-t, *J* = 9.6 Hz, 1 H), 4.80 (pseudo-t, *J* = 9.0 Hz, 1 H), 4.69 (d, *J* = 12.0 Hz, 1 H), 4.65 (d, *J* = 11.4 Hz, 1 H), 4.50 (d, *J* = 11.4 Hz, 1 H), 4.49 (d, *J* = 12.0 Hz, 1 H), 4.47 (d, *J* = 11.4 Hz, 1 H), 4.45 (d, *J* = 11.4 Hz, 1 H), 4.43 (d, *J* =12.0 Hz, 1 H), 4.41 (d, *J* = 8.4 Hz, 1 H), 4.41 – 4.39 (m, 3 H), 4.36 (d, *J* = 12.0 Hz, 1 H), 4.32 (d, *J* = 8.4 Hz, 1 H), 4.14 (pseudo-t, *J* = 9.6 Hz, 2 H), 4.10 (pseudo-t, *J* = 9.6 Hz, 1 H), 3.97 (pseudo-t, *J* = 3.0 Hz, 1 H), 3.94 – 3.90 (m, 1 H), 3.87 (pseudo-t, *J* = 9.0 Hz, 1 H), 3.83 (pseudo-t, *J* = 9.0 Hz, 1 H), 3.79 (pseudo-t, *J* = 9.0 Hz, 1 H), 3.65 – 3.49 (m, 8 H), 3.42 (d, *J* = 9.6 Hz, 1 H), 3.37 (pseudo-t, *J* = 9.0 Hz, 1 H), 3.30 (d, *J* = 9.0 Hz, 1 H), 3.24 (d, *J* = 11.4 Hz, 1 H), 3.19 (s, 9 H), 3.09 (d, *J* = 5.4 Hz, 1 H), 1.99 (s, 3 H), 1.97 (s, 3 H), 1.88 (s, 3 H), 1.83 (s, 3 H), 1.74 (s, 3 H), 1.67 (s, 3 H). ^{13}C NMR ($CDCl_3$, 150 MHz) δ 170.9, 170.8, 170.7, 170.1, 170.0, 169.9, 162.7 (*J* = 246.5 Hz), 137.7, 137.6, 137.5, 137.0, 136.4, 136.2, 135.2 (*J* = 7.8 Hz), 128.8, 128.7, 128.6, 128.58, 128.53, 128.48, 128.45, 128.3, 128.1, 128.0, 127.8, 127.7, 127.3 115.8 (*J* = 21.8 Hz), 100.5, 99.9, 93.4, 86.5, 80.3, 78.4, 77.1, 74.6, 74.1, 74.0, 73.7, 73.3, 73.2, 73.0, 72.9, 72.3, 71.7, 71.6, 69.3, 69.2, 68.0, 67.9, 54.2, 54.0, 53.9, 52.5, 29.6, 23.3, 23.2, 23.0, 20.8, 20.7, 20.6. HRMS (ESI) *m/z* calcd for $C_{87}H_{104}FN_4O_{22}S$ $[M]^+$, 1607.6841; found, 1607.6813.

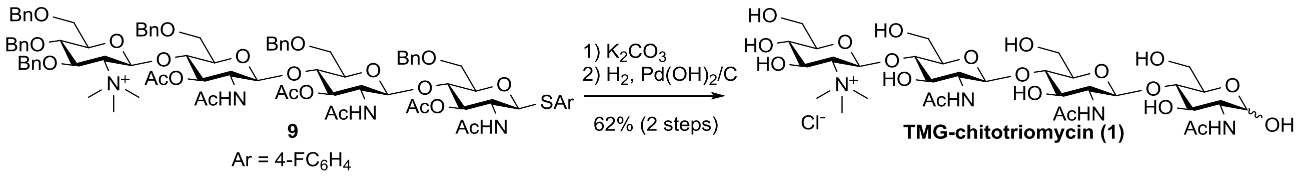

Scheme S5.

Tetrasaccharide **9** (138 mg, 0.086 mmol) was dissolved in a mix-solvent of methanol and CH_2Cl_2 (12 mL/6.0 mL). Then K_2CO_3 (117 mg, 0.86 mmol) was added to the reaction mixture and stirred for 2 h at room temperature. The reaction was quenched by addition of Dowex-50WX4-200 (cation exchange resin) and the resin was removed by filtration. Thus-obtained reaction mixture was evaporated to remove the solvent and the crude product was purified with silica gel chromatography (CH_2Cl_2/MeOH 8:1 as eluent). The product was dissolved in a mix-solvent of water/THF (12.4 mL/3.1 mL) and a few drops of conc. HCl (37%) were added. Then the reaction mixture was cooled by liquid N_2 and degassed under vacuum for 3 times. A catalyst $Pd(OH)_2/C$ (20%) (614 mg) was added to the cooled reaction mixture and filled with hydrogen gas. The sealed container was stirred at room temperature for 12 h. The reaction was quenched with Amberlite® IRN78 (HO^- form) and then Amberlite® IRA402 (Cl^- form) and solvent was removed under reduced pressure to obtain TMG-chiotriomycin (**1**)[2] (44.5 mg, 0.0535 mmol) in 62%

yield (2 steps). Thus-obtained product was checked by ^{1}H-NMR (600 MHz, CD_3OD) and ESI-MS analyses. HRMS (ESI) *m/z* calcd for $C_{33}H_{59}N_4O_{20}$ [M-Cl]$^{+}$, 831.3717; found, 831.3723.

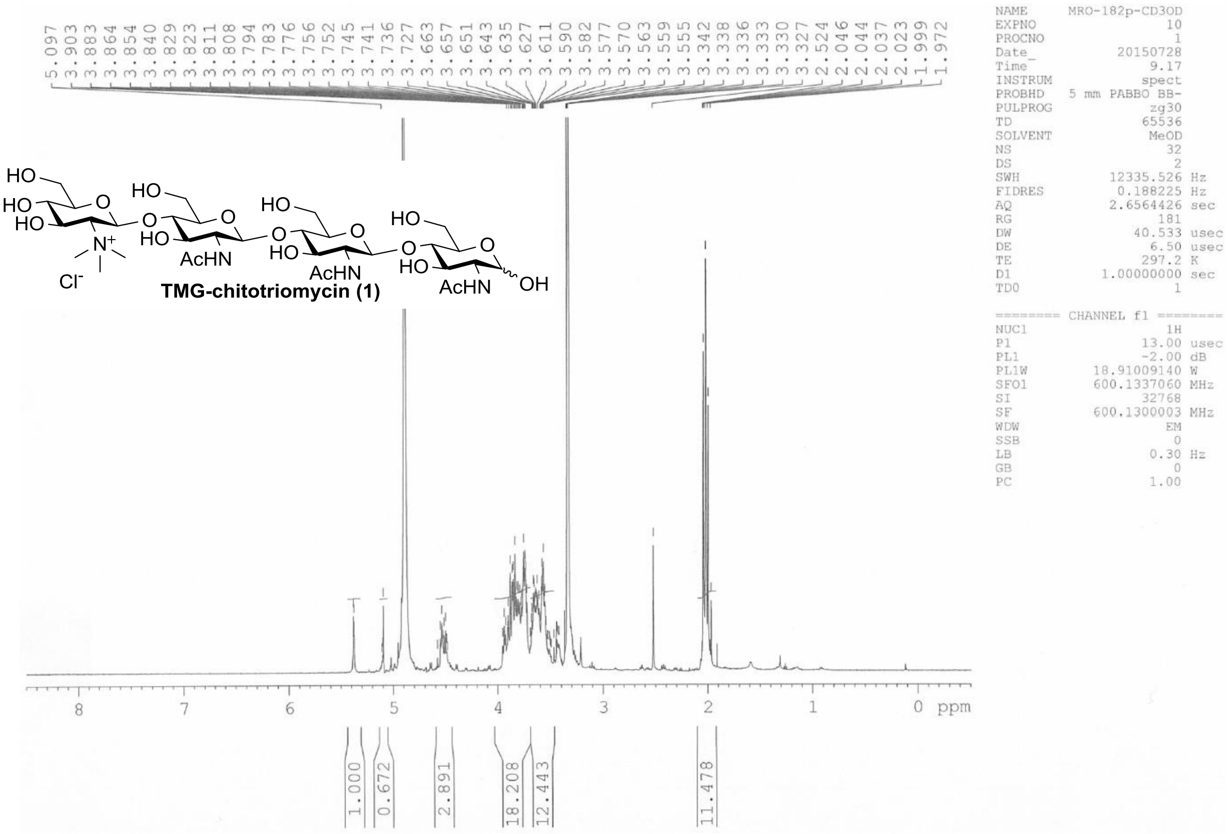

4. References

(1) Nokami, T.; Isoda, Y.; Sasaki, N.; Takaiso, A.; Hayase, S.; Itoh, T.; Hayashi, R.; Shimizu, A.; Yoshida, J. *Org. Lett.* **2015**, *17*, 1525.

(2) Yang, Y.; Li, Y.; Yu, B. *J. Am. Chem. Soc.* **2009**, *131*, 12076.

(3) Despras, G.; Alix, A.; Urban, D.; Vauzeilles, B.; Beau, J.-M. *Angew. Chem. Int. Ed.* **2014**, *53*, 11912.

5. ^{1}H, ^{13}C NMR, H,H-COSY, and HMQC spectra of disaccharides 5aα and 5bα

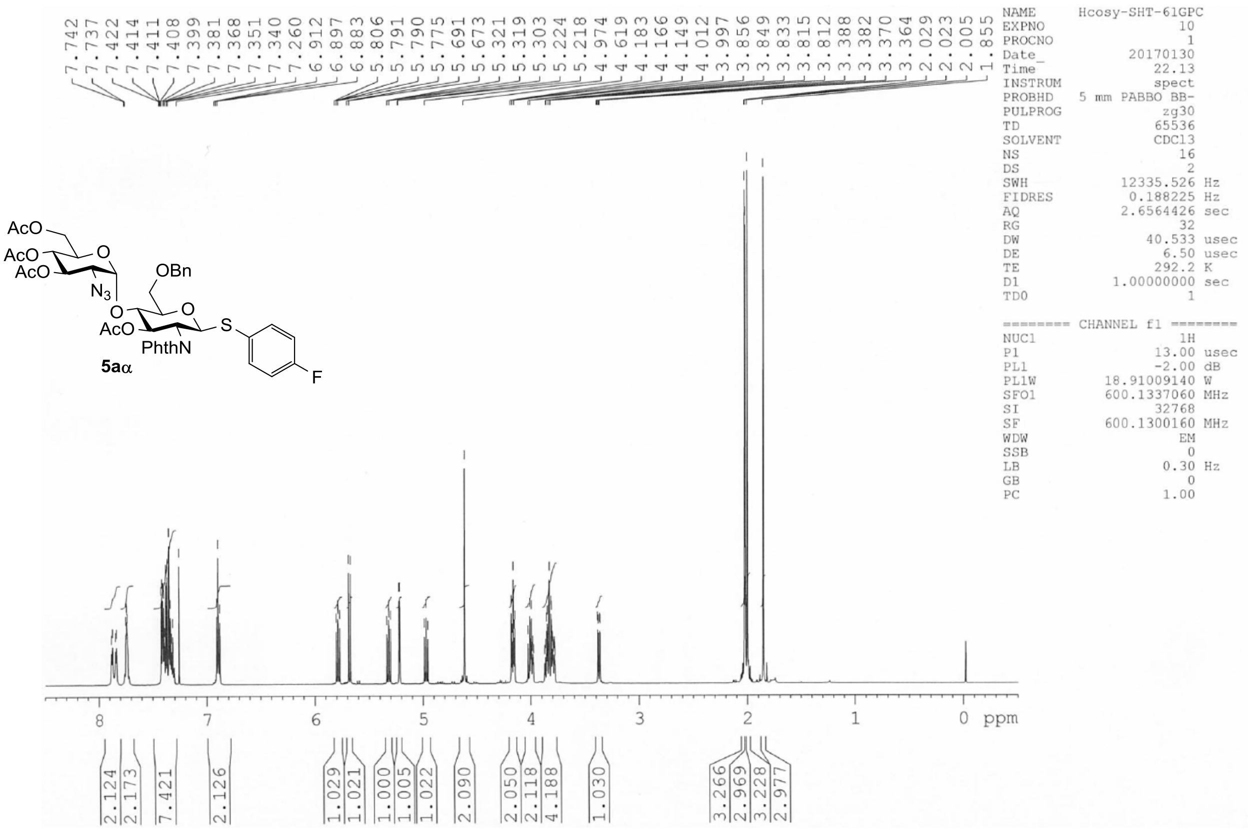

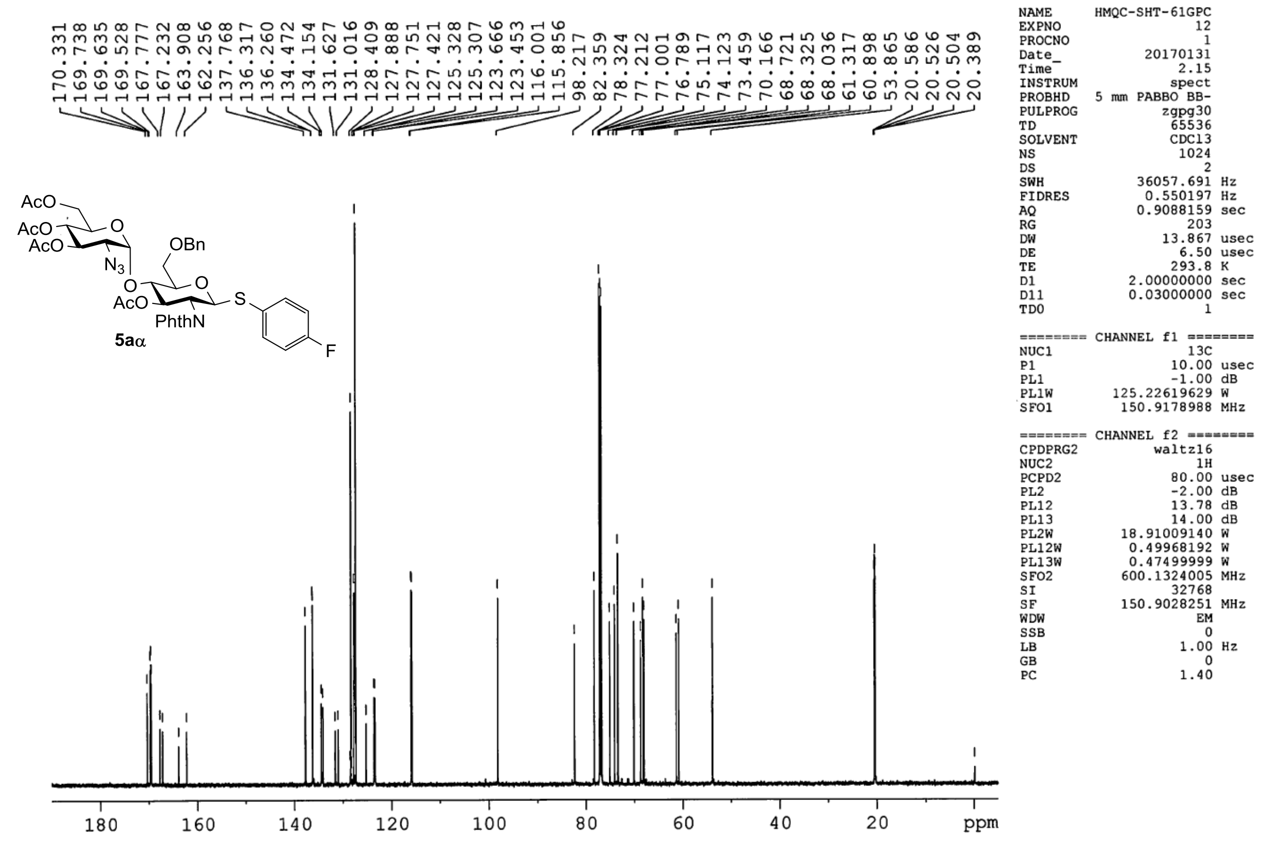

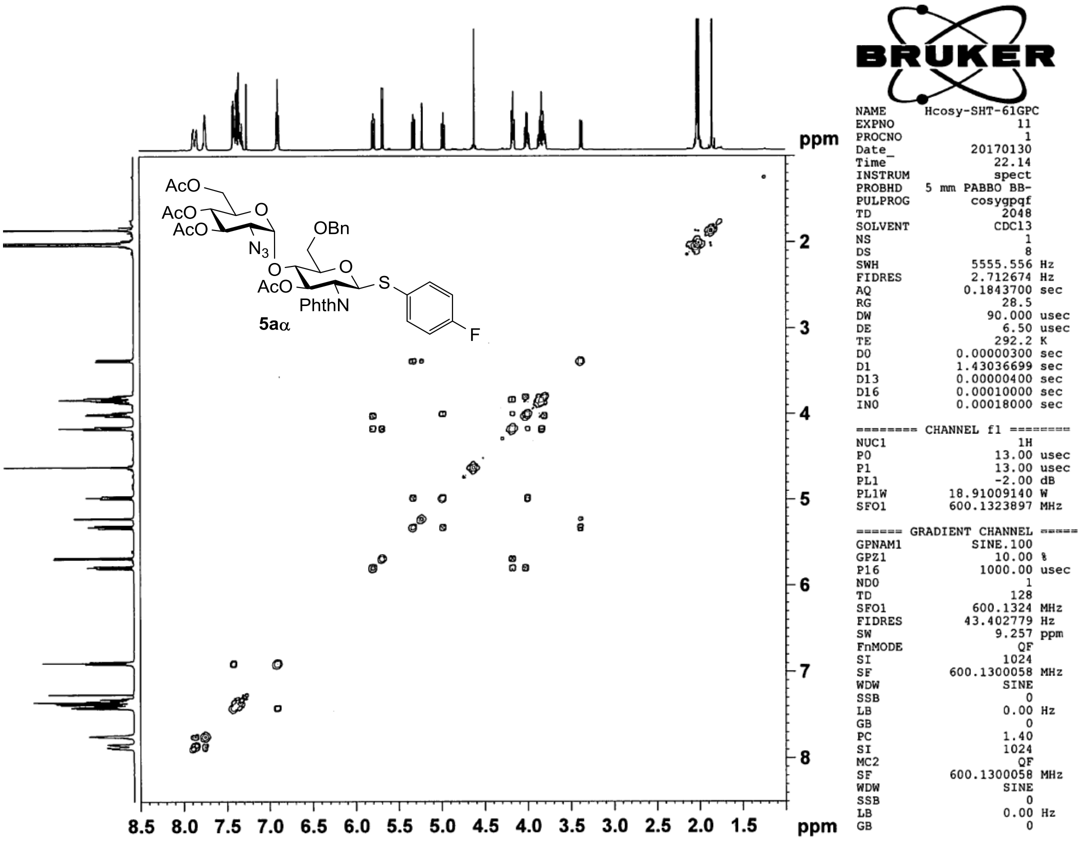
AcO
AcO
AcO
N3
O
OBn
AcO
PhthN
S
F
5aα
ppm
8.5 8.0 7.5 7.0 6.5 6.0 5.5 5.0 4.5 4.0 3.5 3.0 2.5 2.0 1.5
2 3 4 5 6 7 8
BRUKER
NAME Hcosy-SHT-61GPC
EXPNO 11
PROCNO 1
Date_ 20170130
Time 22.14
INSTRUM spect
PROBHD 5 mm PABBO BB-
PULPROG cosygpqf
TD 2048
SOLVENT CDCl3
NS 1
DS 8
SWH 5555.556 Hz
FIDRES 2.712674 Hz
AQ 0.1843700 sec
RG 28.5
DW 90.000 usec
DE 6.50 usec
TE 292.2 K
D0 0.00000300 sec
D1 1.43036699 sec
D13 0.00000400 sec
D16 0.00010000 sec
IN0 0.00018000 sec
======== CHANNEL f1 ========
NUC1 1H
P0 13.00 usec
P1 13.00 usec
PL1 -2.00 dB
PL1W 18.91009140 W
SFO1 600.1323897 MHz
====== GRADIENT CHANNEL =====
GPNAM1 SINE.100
GPZ1 10.00 %
P16 1000.00 usec
ND0 1
TD 128
SFO1 600.1324 MHz
FIDRES 43.402779 Hz
SW 9.257 ppm
FnMODE QF
SI 1024
SF 600.1300058 MHz
WDW SINE
SSB 0
LB 0.00 Hz
GB 0
PC 1.40
SI 1024
MC2 QF
SF 600.1300058 MHz
WDW SINE
SSB 0
LB 0.00 Hz
GB 0

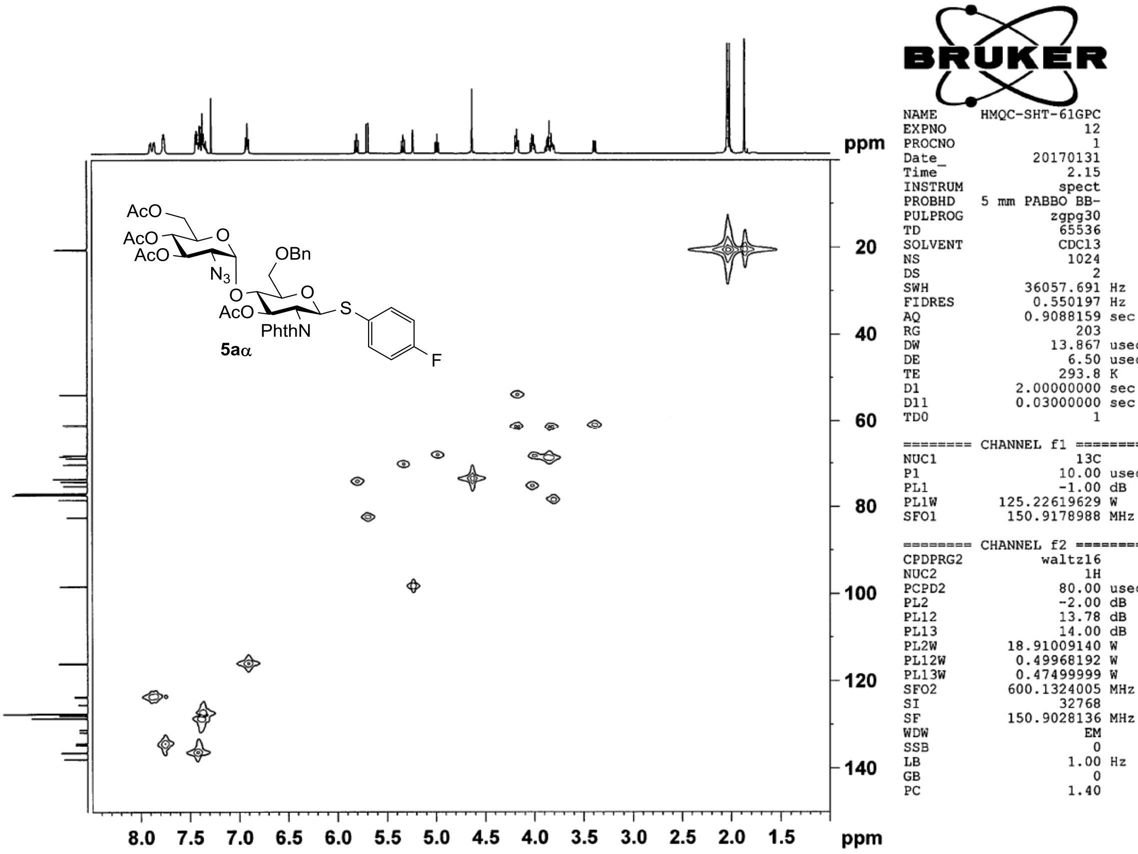
AcO
AcO
AcO
N3
O
OBn
AcO
PhthN
S
F
5aα
ppm
8.0 7.5 7.0 6.5 6.0 5.5 5.0 4.5 4.0 3.5 3.0 2.5 2.0 1.5
20 40 60 80 100 120 140
BRUKER
NAME HMQC-SHT-61GPC
EXPNO 12
PROCNO 1
Date_ 20170131
Time 2.15
INSTRUM spect
PROBHD 5 mm PABBO BB-
PULPROG zgpg30
TD 65536
SOLVENT CDCl3
NS 1024
DS 2
SWH 36057.691 Hz
FIDRES 0.550197 Hz
AQ 0.9088159 sec
RG 203
DW 13.867 usec
DE 6.50 usec
TE 293.8 K
D1 2.00000000 sec
D11 0.03000000 sec
TD0 1
======== CHANNEL f1 ========
NUC1 13C
P1 10.00 usec
PL1 -1.00 dB
PL1W 125.22619629 W
SFO1 150.9178988 MHz
======== CHANNEL f2 ========
CPDPRG2 waltz16
NUC2 1H
PCPD2 80.00 usec
PL2 -2.00 dB
PL12 13.78 dB
PL13 14.00 dB
PL2W 18.91009140 W
PL12W 0.49968192 W
PL13W 0.47499999 W
SFO2 600.1324005 MHz
SI 32768
SF 150.9028136 MHz
WDW EM
SSB 0
LB 1.00 Hz
GB 0
PC 1.40

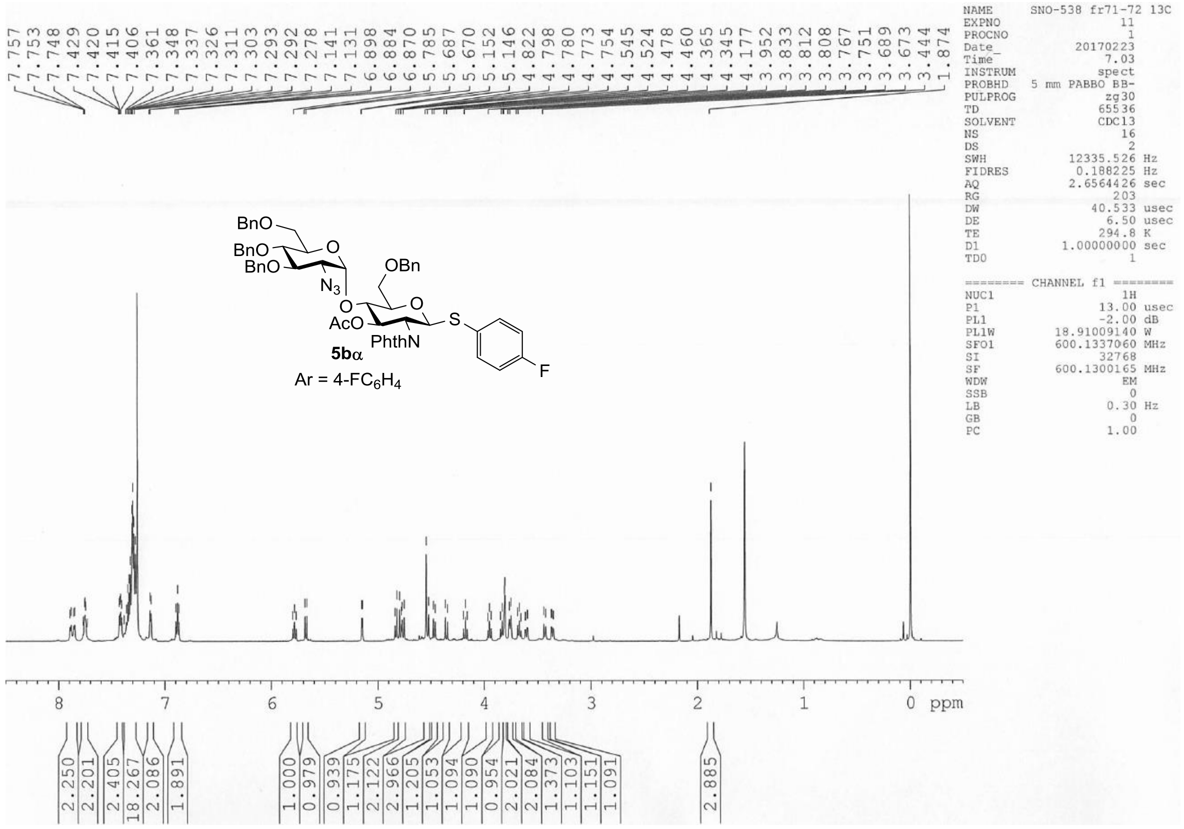
7.757 7.753 7.748 7.429 7.420 7.415 7.406 7.361 7.348 7.337 7.326 7.311 7.303 7.293 7.292 7.278 7.141 7.131 6.898 6.884 6.870 5.785 5.687 5.670 5.152 5.146 4.822 4.798 4.780 4.773 4.754 4.545 4.524 4.478 4.460 4.365 4.345 4.177 3.952 3.833 3.812 3.808 3.767 3.751 3.689 3.673 3.444 1.874
NAME SNO-538 fr71-72 13C
EXPNO 11
PROCNO 1
Date_ 20170223
Time 7.03
INSTRUM spect
PROBHD 5 mm PABBO BB-
PULPROG zg30
TD 65536
SOLVENT CDCl3
NS 16
DS 2
SWH 12335.526 Hz
FIDRES 0.188225 Hz
AQ 2.6564426 sec
RG 203
DW 40.533 usec
DE 6.50 usec
TE 294.8 K
D1 1.00000000 sec
TD0 1
======== CHANNEL f1 ========
NUC1 1H
P1 13.00 usec
PL1 -2.00 dB
PL1W 18.91009140 W
SFO1 600.1337060 MHz
SI 32768
SF 600.1300165 MHz
WDW EM
SSB 0
LB 0.30 Hz
GB 0
PC 1.00
BnO
BnO
BnO
O
N3
OBn
O
O
AcO
PhthN
S
F
5bα
Ar = 4-FC6H4
8 7 6 5 4 3 2 1 0 ppm
2.250 2.201 2.405 18.267 2.086 1.891 1.000 0.979 0.939 1.175 2.122 2.966 1.205 1.053 1.094 1.090 0.954 2.021 2.084 1.373 1.103 1.151 1.091 2.885

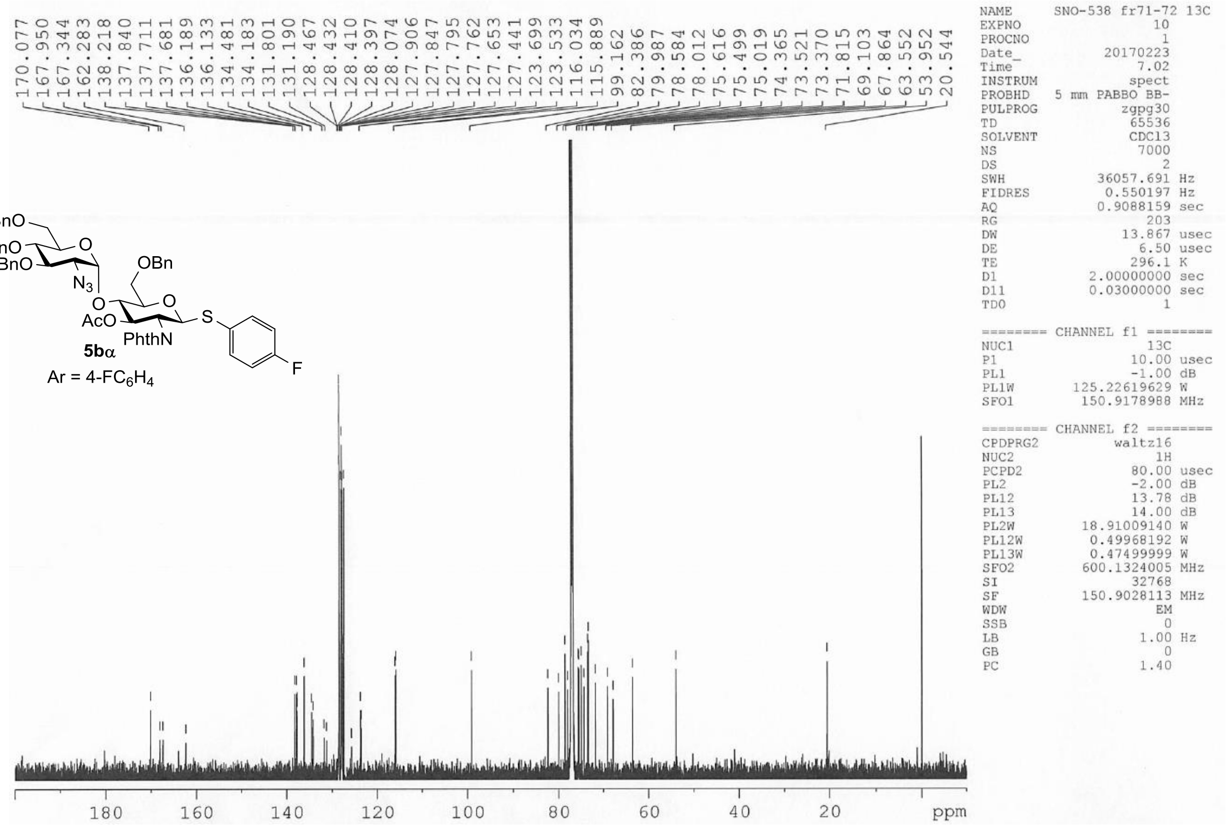
170.077 167.950 167.344 162.283 138.218 137.840 137.711 137.681 136.189 136.133 134.481 134.183 131.801 131.190 128.467 128.432 128.410 128.397 128.074 127.906 127.847 127.795 127.762 127.653 127.441 123.699 123.533 116.034 115.889 99.162 82.386 79.987 78.584 78.012 75.616 75.499 75.019 74.365 73.521 73.370 71.815 69.103 67.864 63.552 53.952 20.544
NAME SNO-538 fr71-72 13C
EXPNO 10
PROCNO 1
Date_ 20170223
Time 7.02
INSTRUM spect
PROBHD 5 mm PABBO BB-
PULPROG zgpg30
TD 65536
SOLVENT CDCl3
NS 7000
DS 2
SWH 36057.691 Hz
FIDRES 0.550197 Hz
AQ 0.9088159 sec
RG 203
DW 13.867 usec
DE 6.50 usec
TE 296.1 K
D1 2.00000000 sec
D11 0.03000000 sec
TD0 1
======== CHANNEL f1 ========
NUC1 13C
P1 10.00 usec
PL1 -1.00 dB
PL1W 125.22619629 W
SFO1 150.9178988 MHz
======== CHANNEL f2 ========
CPDPRG2 waltz16
NUC2 1H
PCPD2 80.00 usec
PL2 -2.00 dB
PL12 13.78 dB
PL13 14.00 dB
PL2W 18.91009140 W
PL12W 0.49968192 W
PL13W 0.47499999 W
SFO2 600.1324005 MHz
SI 32768
SF 150.9028113 MHz
WDW EM
SSB 0
LB 1.00 Hz
GB 0
PC 1.40
BnO
BnO
BnO
O
N3
OBn
O
O
AcO
PhthN
S
F
5bα
Ar = 4-FC6H4
180 160 140 120 100 80 60 40 20 ppm

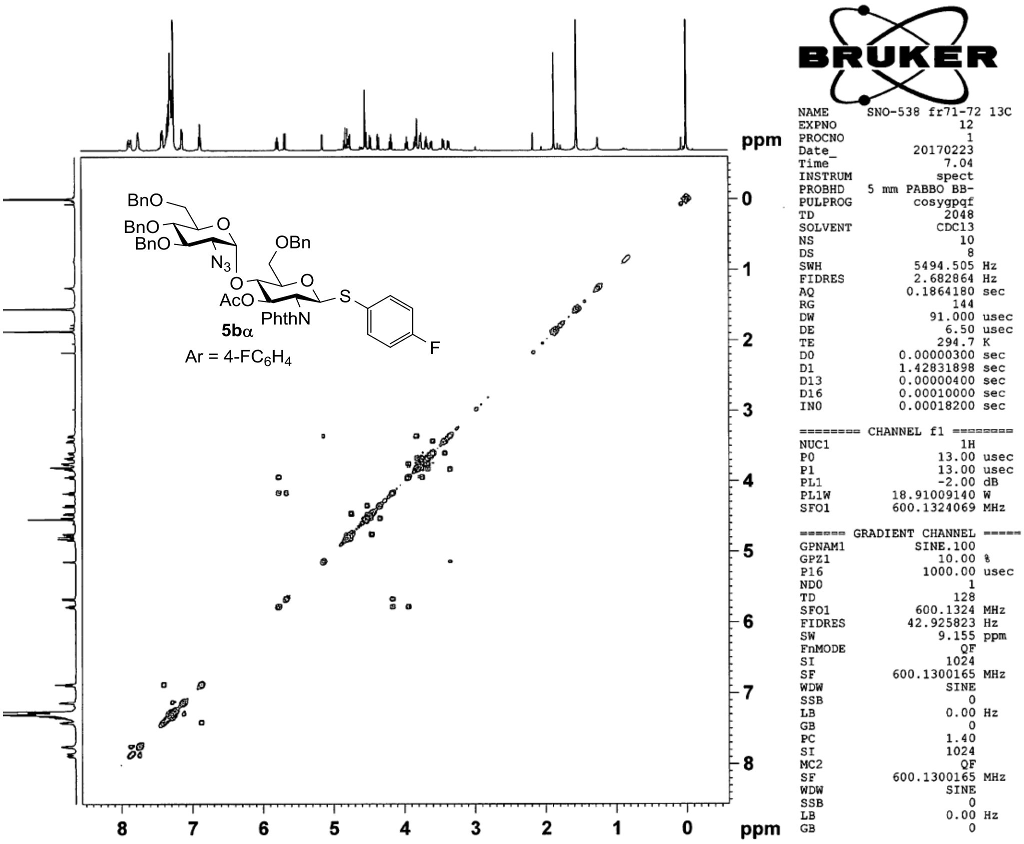
BnO
BnO
BnO
O
N3
O
OBn
O
AcO
PhthN
S
F
5bα
Ar = 4-FC6H4
ppm
NAME SNO-538 fr71-72 13C
EXPNO 12
PROCNO 1
Date_ 20170223
Time 7.04
INSTRUM spect
PROBHD 5 mm PABBO BB-
PULPROG cosygpqf
TD 2048
SOLVENT CDCl3
NS 10
DS 8
SWH 5494.505 Hz
FIDRES 2.682864 Hz
AQ 0.1864180 sec
RG 144
DW 91.000 usec
DE 6.50 usec
TE 294.7 K
D0 0.00000300 sec
D1 1.42831898 sec
D13 0.00000400 sec
D16 0.00010000 sec
IN0 0.00018200 sec
======== CHANNEL f1 ========
NUC1 1H
P0 13.00 usec
P1 13.00 usec
PL1 -2.00 dB
PL1W 18.91009140 W
SFO1 600.1324069 MHz
====== GRADIENT CHANNEL =====
GPNAM1 SINE.100
GPZ1 10.00 %
P16 1000.00 usec
ND0 1
TD 128
SFO1 600.1324 MHz
FIDRES 42.925823 Hz
SW 9.155 ppm
FnMODE QF
SI 1024
SF 600.1300165 MHz
WDW SINE
SSB 0
LB 0.00 Hz
GB 0
PC 1.40
SI 1024
MC2 QF
SF 600.1300165 MHz
WDW SINE
SSB 0
LB 0.00 Hz
GB 0

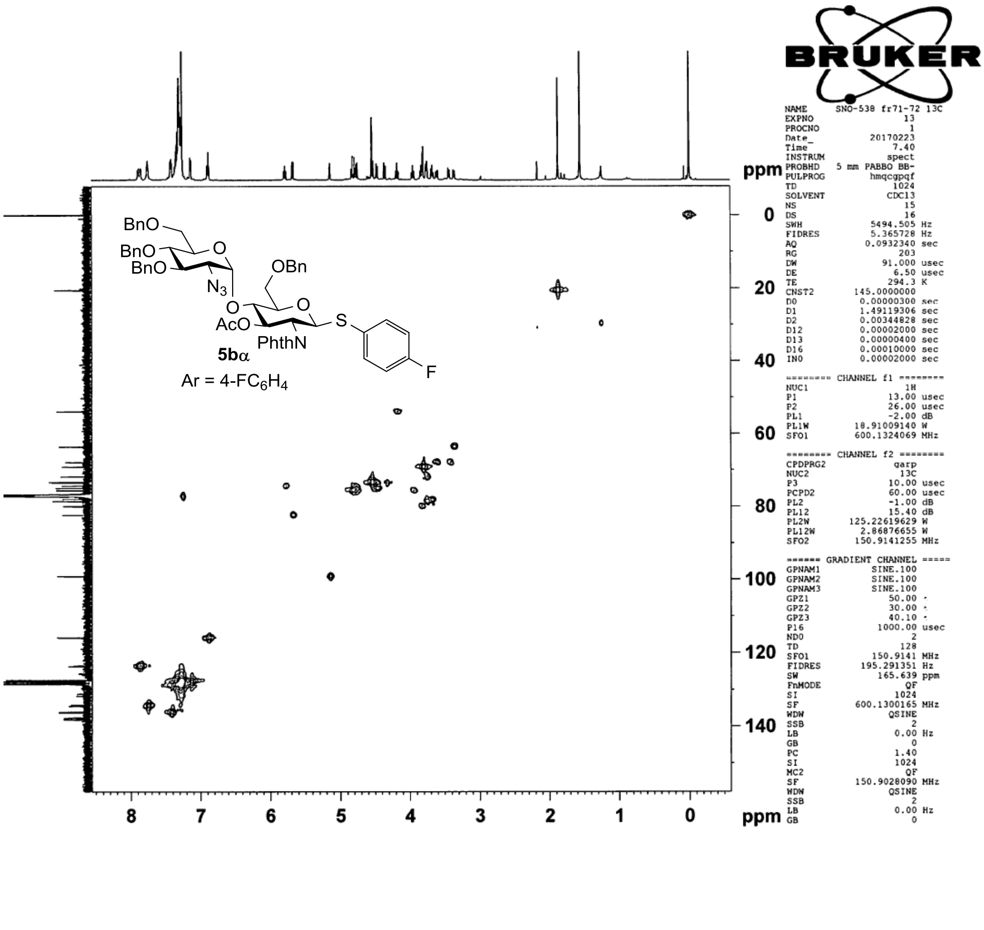
BnO
BnO
BnO
O
N3
O
OBn
O
AcO
PhthN
S
F
5bα
Ar = 4-FC6H4
ppm
NAME SNO-538 fr71-72 13C
EXPNO 13
PROCNO 1
Date_ 20170223
Time 7.40
INSTRUM spect
PROBHD 5 mm PABBO BB-
PULPROG hmqcgpqf
TD 1024
SOLVENT CDCl3
NS 15
DS 16
SWH 5494.505 Hz
FIDRES 5.365728 Hz
AQ 0.0932340 sec
RG 203
DW 91.000 usec
DE 6.50 usec
TE 294.3 K
CNST2 145.0000000
D0 0.00000300 sec
D1 1.49119306 sec
D2 0.00344828 sec
D12 0.00002000 sec
D13 0.00000400 sec
D16 0.00010000 sec
IN0 0.00002000 sec
======== CHANNEL f1 ========
NUC1 1H
P1 13.00 usec
P2 26.00 usec
PL1 -2.00 dB
PL1W 18.91009140 W
SFO1 600.1324069 MHz
======== CHANNEL f2 ========
CPDPRG2 garp
NUC2 13C
P3 10.00 usec
PCPD2 60.00 usec
PL2 -1.00 dB
PL12 15.40 dB
PL2W 125.22619629 W
PL12W 2.86876655 W
SFO2 150.9141255 MHz
====== GRADIENT CHANNEL =====
GPNAM1 SINE.100
GPNAM2 SINE.100
GPNAM3 SINE.100
GPZ1 50.00
GPZ2 30.00
GPZ3 40.10
P16 1000.00 usec
ND0 2
TD 128
SFO1 150.9141 MHz
FIDRES 195.291351 Hz
SW 165.639 ppm
FnMODE QF
SI 1024
SF 600.1300165 MHz
WDW QSINE
SSB 2
LB 0.00 Hz
GB 0
PC 1.40
SI 1024
MC2 QF
SF 150.9028090 MHz
WDW QSINE
SSB 2
LB 0.00 Hz
GB 0

6. ^{1}H, ^{13}C-NMR, H,H-COSY, and HMQC spectra of tetrasaccharides 8 and 9

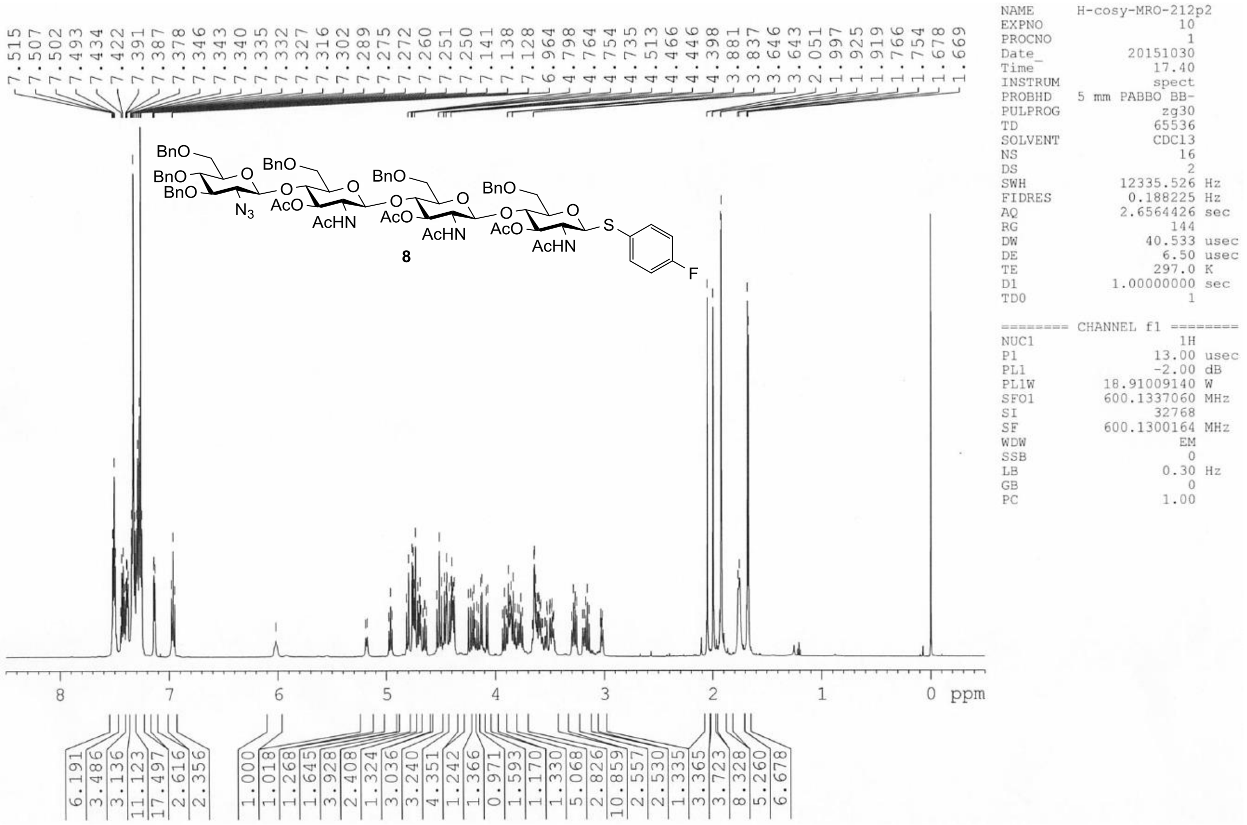

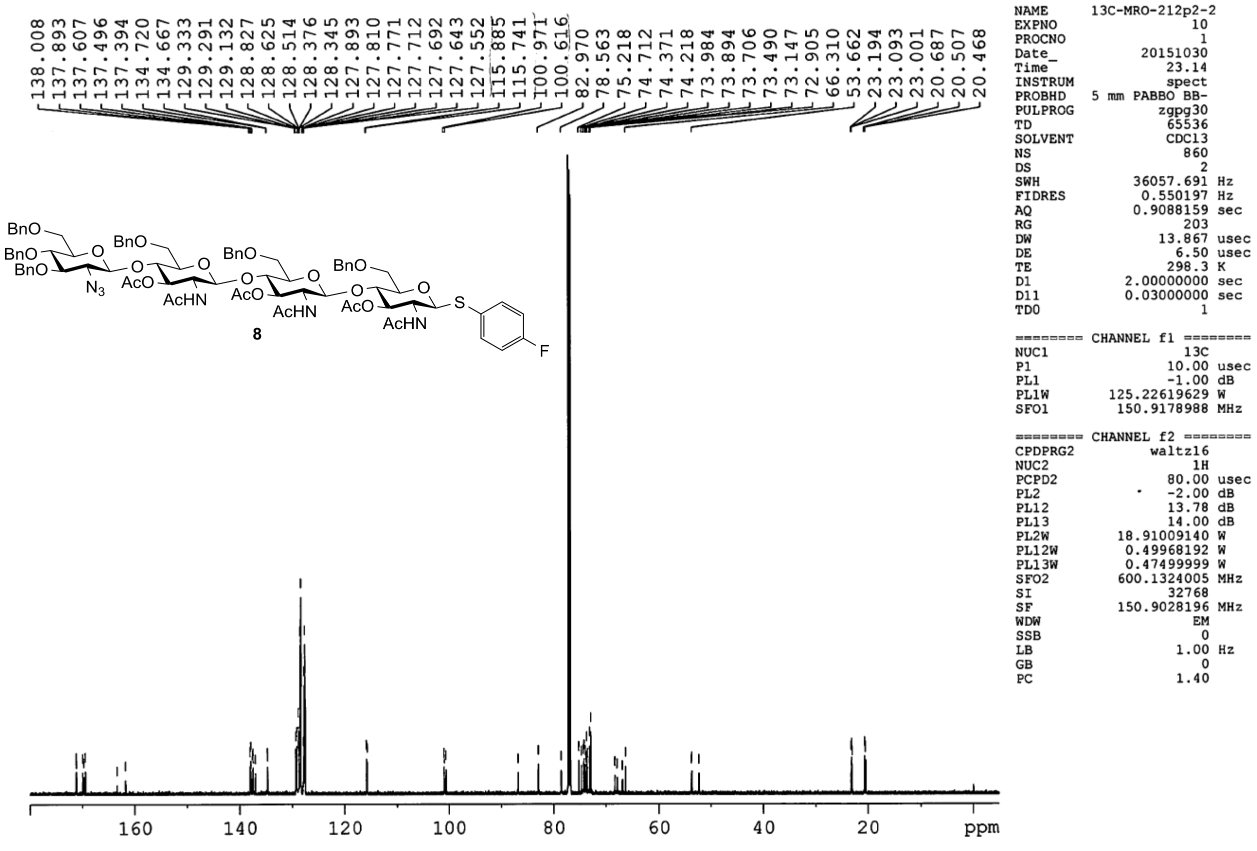

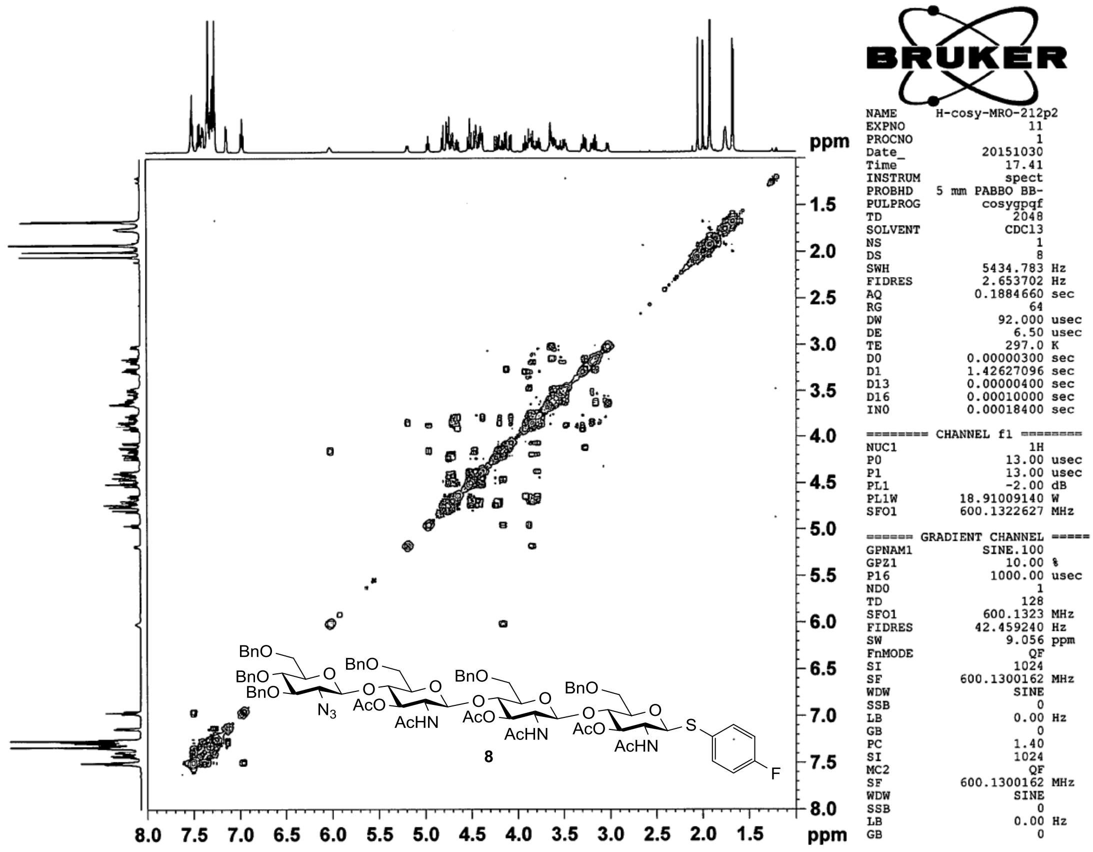
BRUKER
NAME H-cosy-MRO-212p2
EXPNO 11
PROCNO 1
Date_ 20151030
Time 17.41
INSTRUM spect
PROBHD 5 mm PABBO BB-
PULPROG cosygpqf
TD 2048
SOLVENT CDCl3
NS 1
DS 8
SWH 5434.783 Hz
FIDRES 2.653702 Hz
AQ 0.1884660 sec
RG 64
DW 92.000 usec
DE 6.50 usec
TE 297.0 K
D0 0.00000300 sec
D1 1.42627096 sec
D13 0.00000400 sec
D16 0.00010000 sec
IN0 0.00018400 sec
======== CHANNEL f1 ========
NUC1 1H
P0 13.00 usec
P1 13.00 usec
PL1 -2.00 dB
PL1W 18.91009140 W
SFO1 600.1322627 MHz
====== GRADIENT CHANNEL =====
GPNAM1 SINE.100
GPZ1 10.00 %
P16 1000.00 usec
ND0 1
TD 128
SFO1 600.1323 MHz
FIDRES 42.459240 Hz
SW 9.056 ppm
FnMODE QF
SI 1024
SF 600.1300162 MHz
WDW SINE
SSB 0
LB 0.00 Hz
GB 0
PC 1.40
SI 1024
MC2 QF
SF 600.1300162 MHz
WDW SINE
SSB 0
LB 0.00 Hz
GB 0
ppm
BnO
BnO
BnO
N3
AcO
AcHN
AcO
AcHN
AcO
AcHN
S
F
8

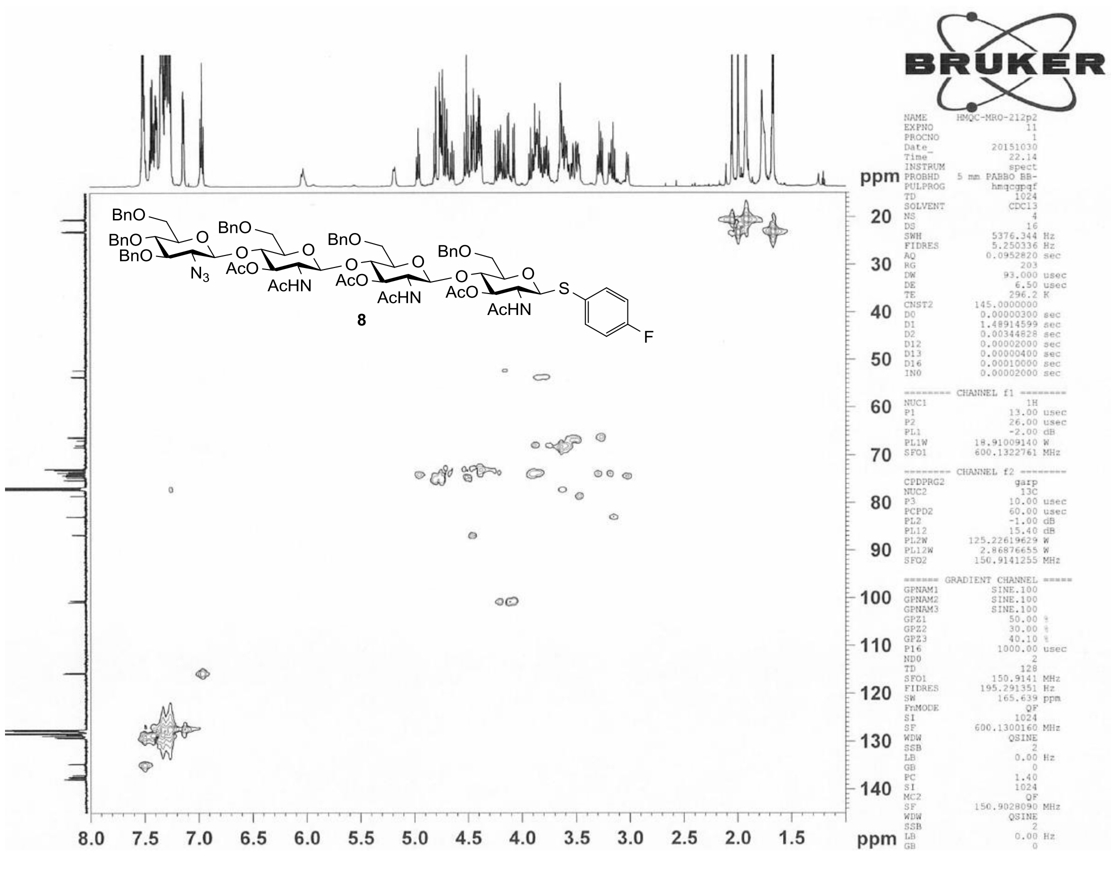
BRUKER
NAME HMQC-MRO-212p2
ppm
BnO
BnO
BnO
N3
AcO
AcHN
AcO
AcHN
AcO
AcHN
S
F
8

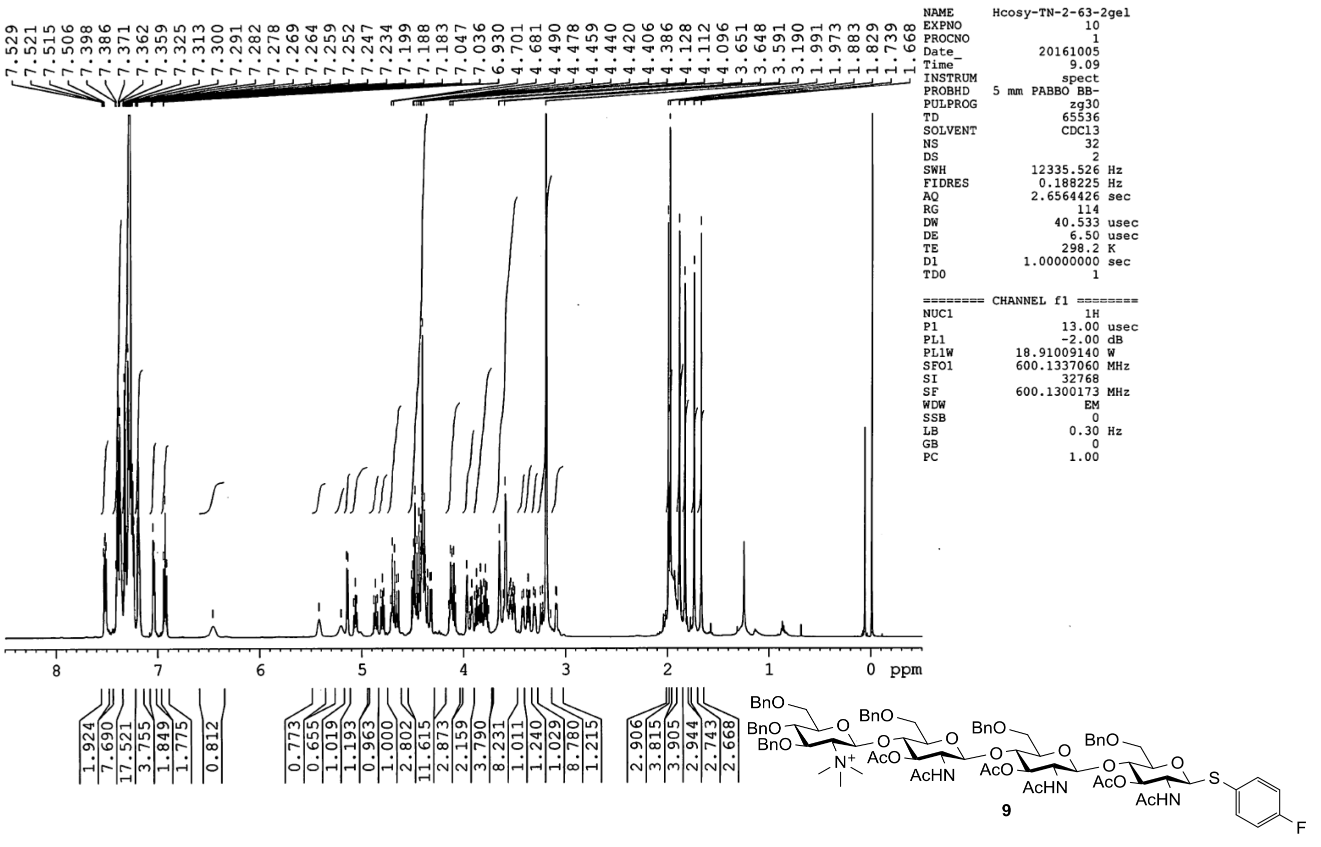
7.529 7.521 7.515 7.506 7.398 7.386 7.371 7.362 7.359 7.325 7.313 7.300 7.291 7.282 7.278 7.269 7.264 7.259 7.252 7.247 7.234 7.199 7.188 7.183 7.047 7.036 6.930 4.701 4.681 4.490 4.478 4.459 4.440 4.420 4.406 4.386 4.128 4.112 4.096 3.651 3.648 3.591 3.190 1.991 1.973 1.883 1.829 1.739 1.668
NAME Hcosy-TN-2-63-2gel
EXPNO 10
PROCNO 1
Date_ 20161005
Time 9.09
INSTRUM spect
PROBHD 5 mm PABBO BB-
PULPROG zg30
TD 65536
SOLVENT CDCl3
NS 32
DS 2
SWH 12335.526 Hz
FIDRES 0.188225 Hz
AQ 2.6564426 sec
RG 114
DW 40.533 usec
DE 6.50 usec
TE 298.2 K
D1 1.00000000 sec
TD0 1
======== CHANNEL f1 ========
NUC1 1H
P1 13.00 usec
PL1 -2.00 dB
PL1W 18.91009140 W
SFO1 600.1337060 MHz
SI 32768
SF 600.1300173 MHz
WDW EM
SSB 0
LB 0.30 Hz
GB 0
PC 1.00
8 7 6 5 4 3 2 1 0 ppm
1.924 7.690 17.521 3.755 1.849 1.775 0.812 0.773 0.655 1.019 1.193 0.963 1.000 2.802 11.615 2.873 2.159 2.159 3.790 8.231 1.011 1.240 1.029 8.780 1.215 2.906 3.815 3.905 2.944 2.743 2.668
BnO BnO BnO BnO BnO BnO BnO
N+
AcO AcO AcO
AcHN AcHN AcHN
O S F
9

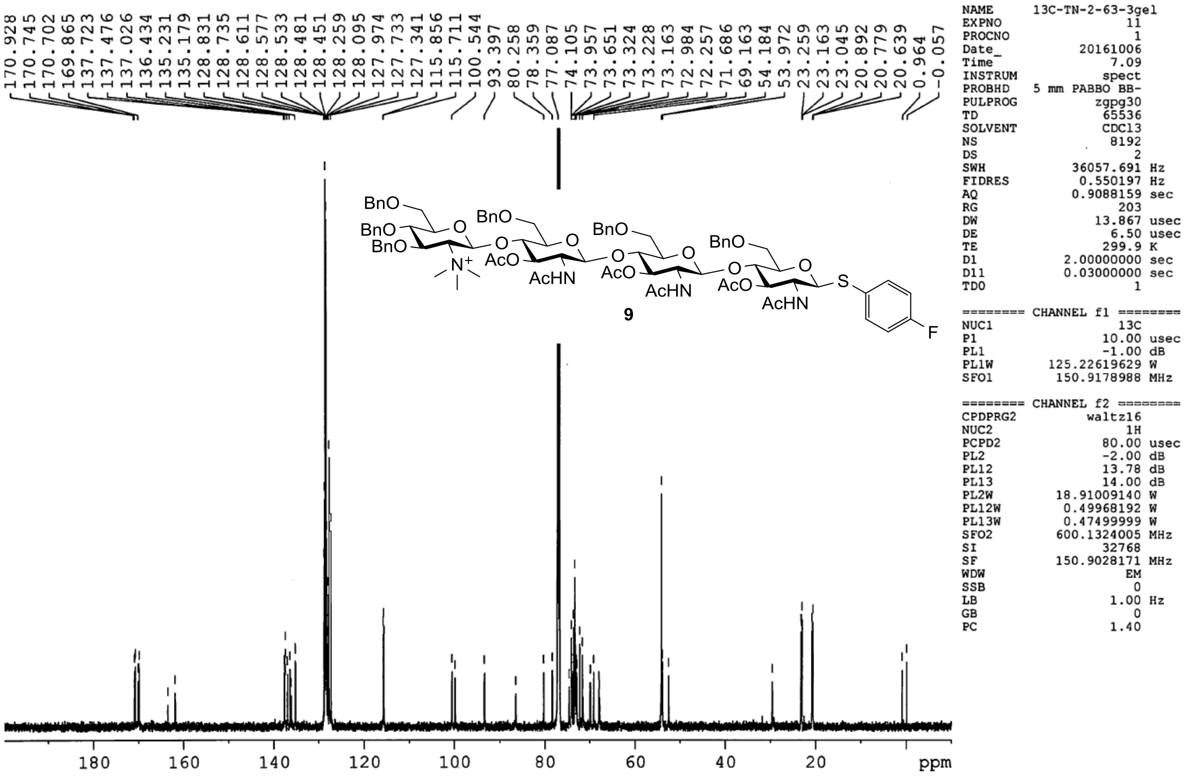
170.928 170.745 170.702 169.865 137.723 137.476 137.026 136.434 135.231 135.179 128.831 128.735 128.611 128.577 128.533 128.481 128.451 128.259 128.095 127.974 127.733 127.341 115.856 115.711 100.544 93.397 80.258 78.359 77.087 74.105 73.957 73.651 73.324 73.228 73.163 72.984 72.257 71.686 69.163 54.184 53.972 23.259 23.163 23.045 20.892 20.779 20.639 0.964 -0.057
NAME 13C-TN-2-63-3gel
EXPNO 11
PROCNO 1
Date_ 20161006
Time 7.09
INSTRUM spect
PROBHD 5 mm PABBO BB-
PULPROG zgpg30
TD 65536
SOLVENT CDCl3
NS 8192
DS 2
SWH 36057.691 Hz
FIDRES 0.550197 Hz
AQ 0.9088159 sec
RG 203
DW 13.867 usec
DE 6.50 usec
TE 299.9 K
D1 2.00000000 sec
D11 0.03000000 sec
TD0 1
======== CHANNEL f1 ========
NUC1 13C
P1 10.00 usec
PL1 -1.00 dB
PL1W 125.22619629 W
SFO1 150.9178988 MHz
======== CHANNEL f2 ========
CPDPRG2 waltz16
NUC2 1H
PCPD2 80.00 usec
PL2 -2.00 dB
PL12 13.78 dB
PL13 14.00 dB
PL2W 18.91009140 W
PL12W 0.49968192 W
PL13W 0.47499999 W
SFO2 600.1324005 MHz
SI 32768
SF 150.9028171 MHz
WDW EM
SSB 0
LB 1.00 Hz
GB 0
PC 1.40
BnO BnO BnO BnO BnO BnO BnO
N+
AcO AcO AcO
AcHN AcHN AcHN
O S F
9
180 160 140 120 100 80 60 40 20 ppm

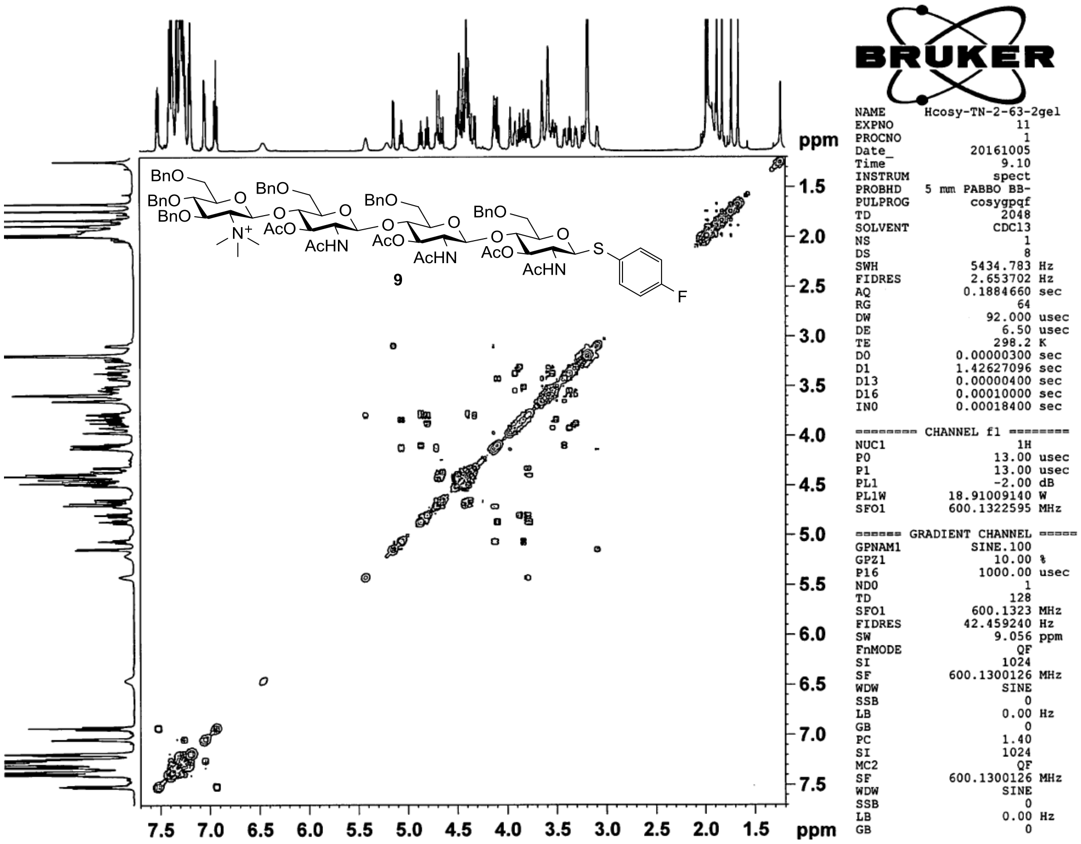
BnO
BnO
BnO
O
N+
AcO
AcHN
S
F
9
ppm
NAME Hcosy-TN-2-63-2gel
EXPNO 11
PROCNO 1
Date_ 20161005
Time 9.10
INSTRUM spect
PROBHD 5 mm PABBO BB-
PULPROG cosygpqf
TD 2048
SOLVENT CDCl3
NS 1
DS 8
SWH 5434.783 Hz
FIDRES 2.653702 Hz
AQ 0.1884660 sec
RG 64
DW 92.000 usec
DE 6.50 usec
TE 298.2 K
D0 0.00000300 sec
D1 1.42627096 sec
D13 0.00000400 sec
D16 0.00010000 sec
IN0 0.00018400 sec
======== CHANNEL f1 ========
NUC1 1H
P0 13.00 usec
P1 13.00 usec
PL1 -2.00 dB
PL1W 18.91009140 W
SFO1 600.1322595 MHz
====== GRADIENT CHANNEL =====
GPNAM1 SINE.100
GPZ1 10.00 %
P16 1000.00 usec
ND0 1
TD 128
SFO1 600.1323 MHz
FIDRES 42.459240 Hz
SW 9.056 ppm
FnMODE QF
SI 1024
SF 600.1300126 MHz
WDW SINE
SSB 0
LB 0.00 Hz
GB 0
PC 1.40
SI 1024
MC2 QF
SF 600.1300126 MHz
WDW SINE
SSB 0
LB 0.00 Hz
GB 0

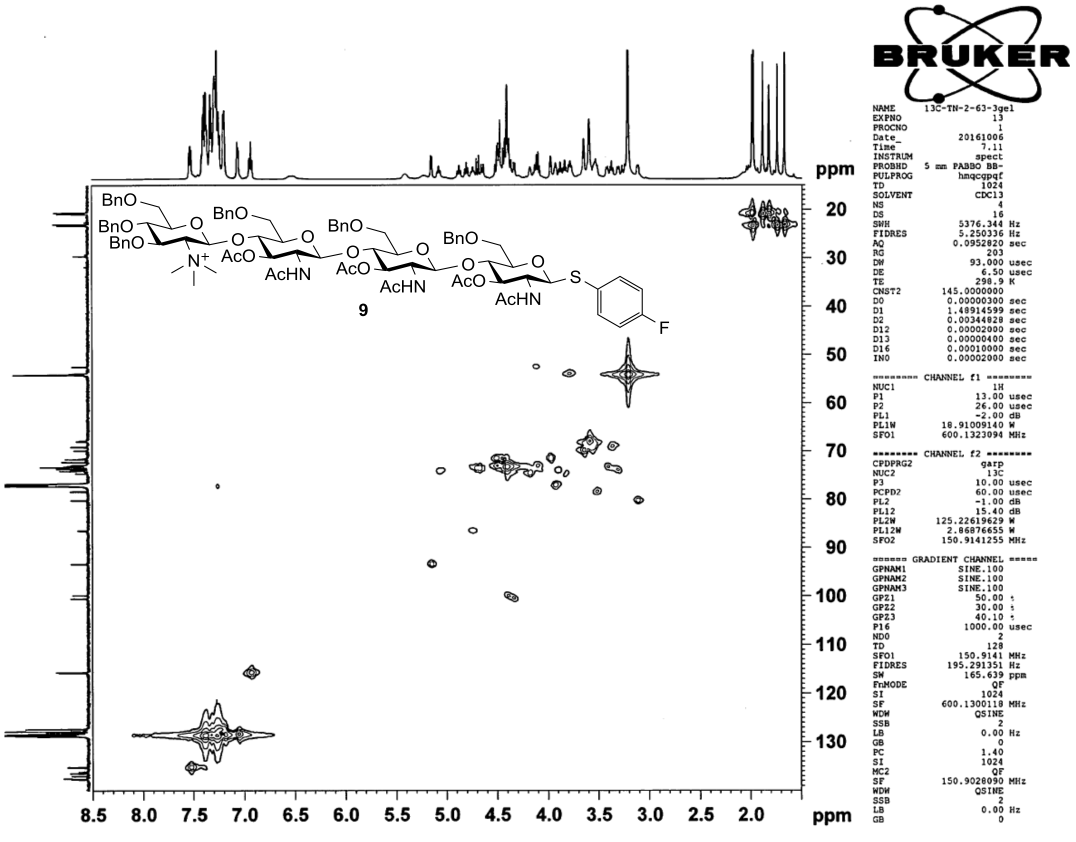
BnO
BnO
BnO
O
N+
AcO
AcHN
S
F
9
ppm
NAME 13C-TN-2-63-3gel
EXPNO 13
PROCNO 1
Date_ 20161006
Time 7.11
INSTRUM spect
PROBHD 5 mm PABBO BB-
PULPROG hmqcgpqf
TD 1024
SOLVENT CDCl3
NS 4
DS 16
SWH 5376.344 Hz
FIDRES 5.250336 Hz
AQ 0.0952820 sec
RG 203
DW 93.000 usec
DE 6.50 usec
TE 298.9 K
CNST2 145.0000000
D0 0.00000300 sec
D1 1.48914599 sec
D2 0.00344828 sec
D12 0.00002000 sec
D13 0.00000400 sec
D16 0.00010000 sec
IN0 0.00002000 sec
======== CHANNEL f1 ========
NUC1 1H
P1 13.00 usec
P2 26.00 usec
PL1 -2.00 dB
PL1W 18.91009140 W
SFO1 600.1323094 MHz
======== CHANNEL f2 ========
CPDPRG2 garp
NUC2 13C
P3 10.00 usec
PCPD2 60.00 usec
PL2 -1.00 dB
PL12 15.40 dB
PL2W 125.22619629 W
PL12W 2.86876655 W
SFO2 150.9141255 MHz
====== GRADIENT CHANNEL =====
GPNAM1 SINE.100
GPNAM2 SINE.100
GPNAM3 SINE.100
GPZ1 50.00 %
GPZ2 30.00 %
GPZ3 40.10 %
P16 1000.00 usec
ND0 2
TD 128
SFO1 150.9141 MHz
FIDRES 195.291351 Hz
SW 165.639 ppm
FnMODE QF
SI 1024
SF 600.1300118 MHz
WDW QSINE
SSB 2
LB 0.00 Hz
GB 0
PC 1.40
SI 1024
MC2 QF
SF 150.9028090 MHz
WDW QSINE
SSB 2
LB 0.00 Hz
GB 0
